# Advancing fluorescence imaging: enhanced control of cyanine dye-doped silica nanoparticles

**DOI:** 10.1186/s12951-024-02638-7

**Published:** 2024-06-19

**Authors:** Taewoong Son, Minseo Kim, Minsuk Choi, Sang Hwan Nam, Ara Yoo, Hyunseung Lee, Eun Hee Han, Kwan Soo Hong, Hye Sun Park

**Affiliations:** 1 Biopharmaceutical Research Center, Ochang Institute of Biological and Environmental Science, Korea Basic Science Institute (KBSI), Cheongju, 28119 Republic of Korea; 2 Graduate School of Analytical Science and Technology (GRAST), Chungnam National University, Daejeon, 34134 Republic of Korea; 3 Laboratory of Nanophotonics & Nanospectroscopic Imaging, Korea Research Institute of Chemical Technology, Daejeon, 34114 Republic of Korea; 4 Department of Chemistry, Sungkyunkwan University, Suwon, 16419 Republic of Korea; 5 Korea University of Science and Technology (UST), Daejeon, 34113 Republic of Korea; 6 Department of Chemistry, Chung-Ang University, Seoul, 06974 Republic of Korea

**Keywords:** Cyanine N-hydroxysuccinimide ester, Silica nanoparticle, Characterization, Fluorescence in vitro and in vivo image, Imaging optimization

## Abstract

**Background:**

Silica nanoparticles (SNPs) have immense potential in biomedical research, particularly in drug delivery and imaging applications, owing to their stability and minimal interactions with biological entities such as tissues or cells.

**Results:**

With synthesized and characterized cyanine-dye-doped fluorescent SNPs (CSNPs) using cyanine 3.5, 5.5, and 7 (Cy3.5, Cy5.5, and Cy7). Through systematic analysis, we discerned variations in the surface charge and fluorescence properties of the nanoparticles contingent on the encapsulated dye-(3-aminopropyl)triethoxysilane conjugate, while their size and shape remained constant. The fluorescence emission spectra exhibited a redshift correlated with increasing dye concentration, which was attributed to cascade energy transfer and self-quenching effects. Additionally, the fluorescence signal intensity showed a linear relationship with the particle concentration, particularly at lower dye equivalents, indicating a robust performance suitable for imaging applications. In vitro assessments revealed negligible cytotoxicity and efficient cellular uptake of the nanoparticles, enabling long-term tracking and imaging. Validation through in vivo imaging in mice underscored the versatility and efficacy of CSNPs, showing single-switching imaging capabilities and linear signal enhancement within subcutaneous tissue environment.

**Conclusions:**

This study provides valuable insights for designing fluorescence imaging and optimizing nanoparticle-based applications in biomedical research, with potential implications for targeted drug delivery and in vivo imaging of tissue structures and organs.

**Graphical Abstract:**

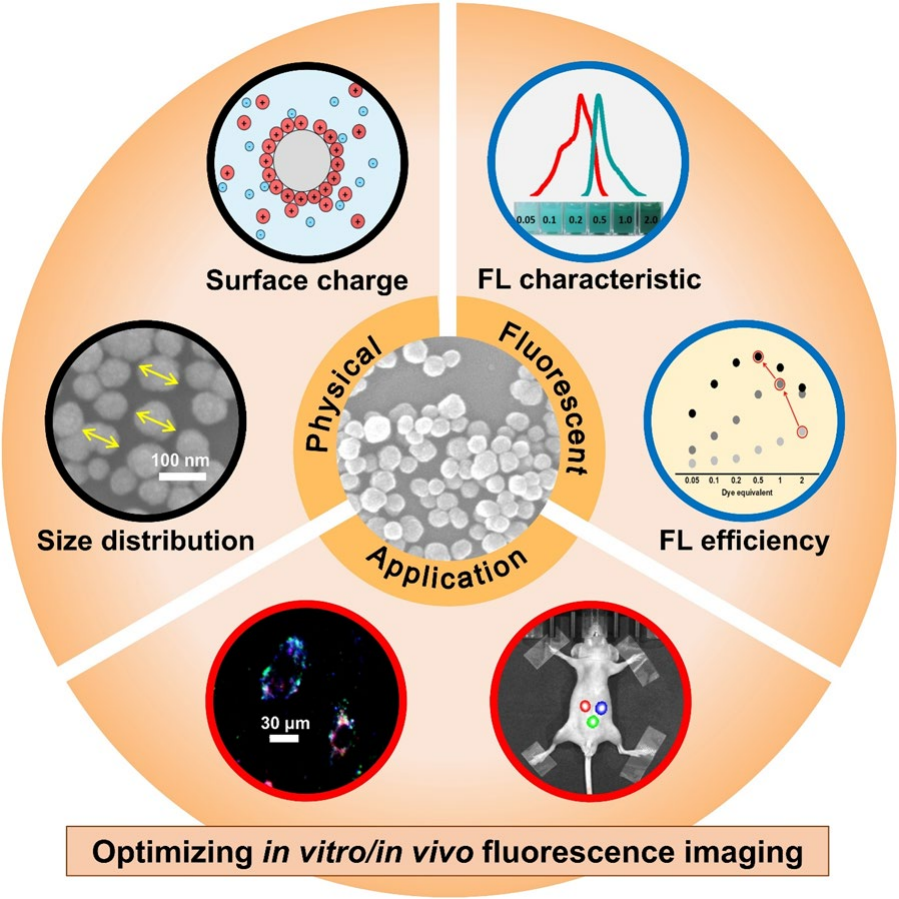

**Supplementary Information:**

The online version contains supplementary material available at 10.1186/s12951-024-02638-7.

## Introduction

A wide range of fluorescent nanoparticles have found widespread application in bioimaging owing to their inherent fluorescent properties and nanoscale material characteristics. These nanoparticles, including semiconductor quantum dots, carbon nanoparticles, noble-metal nanoparticles, and silica nanoparticles (SNPs), have been extensively used in disease diagnosis, therapeutic interventions, and functional investigations at the cellular level, leveraging diverse particle morphologies and surface modifications. Nevertheless, considerations such as fluorescence stability, biocompatibility, manufacturing versatility, and application flexibility offer researchers a spectrum of options in pertinent fields [1].

SNPs have emerged as a focal point of research in medicine and life sciences because of their unique physical and chemical properties, and diverse potential applications [2, 3]. Notably, their inherent stability and minimal interactions with tissues and cells make them promising candidates as multifunctional carriers in various medical fields, including diagnosis, sensing, and drug delivery [4]. These nanoparticles are versatile and useful for tumor diagnosis, cardiovascular disease treatment, and tumor thermotherapy [2, 5, 6].

The Stöber method is a prominent approach for synthesizing SNPs, which offers precise control over their size and structure by manipulating the ammonium solution concentration [7]. The method also facilitates the enhancement of stability and reduction of interactions with biological entities through the core–shell coating [8–10]. Consequently, SNPs are versatile platforms for drug delivery and imaging sensors, offering a high loading capacity and controlled release mechanisms for biological drugs [4, 11–13].

Fluorescent-dye-conjugated SNPs play a pivotal role in research, enabling visual labeling and tracking within biological frameworks [3, 14]. Leveraging optical methodologies within the near-infrared spectrum in the wavelength range 650–900 nm optimizes the sensitivity for imaging deep-seated tissue structures, thus utilizing the inherent translucency of biological tissues and mitigating challenges related to scattering and absorption [15]. Moreover, because each nanoparticle can accommodate thousands of dye molecules, it provides stable average signal values from numerous encapsulated dye molecules [3], offering a non-invasive technique with remarkable sensitivity and specificity [16, 17]. This synergistic fusion of advantages provides researchers with clear “visual insights”, driving the exploration of both research and therapeutic applications [14].

This study investigated the physical and fluorescence properties of plain SNPs and cyanine-dye-doped fluorescent SNPs (CSNPs) with various loading equivalents. Using cyanine dyes coupled to SNPs via (3-aminopropyl)triethoxysilane (APTES) bonding [18, 19], we explored the impact of dye loading on particle properties. In addition, SNPs were fabricated to observe the changes in physical properties induced by the dye. SNPs were synthesized using the same protocol as that for CSNPs, with the only difference being the exclusion of dye from the premixture solution. Six particle types were synthesized using varying amounts of APTES. By analyzing the particle size distribution, surface zeta potential, and spectral characteristics of the SNPs and CSNPs, we discerned notable changes in the nanoparticle behavior based on the synthesis conditions.

Tailored SNPs with precise structures and chemical properties hold immense promise for biological and medical applications, facilitating in vivo imaging, targeting, and drug delivery [20, 21]. The demand for narrow size distribution, chemical stability, and hydrophilic dispersibility underscores the significance of SNPs in fluorescence labeling for non-invasive in vivo imaging [22, 23]. By addressing the effect of the amount of loaded fluorescent dye on the properties of SNPs, our study provides guidelines for the production of fluorescent nanoparticles suitable for the purpose of the studies, offering insights to improve imaging performance in diverse applications.

## Results and discussion

### Synthesis of cyanine-dye-doped fluorescent silica nanoparticles

**Fig. 1:**
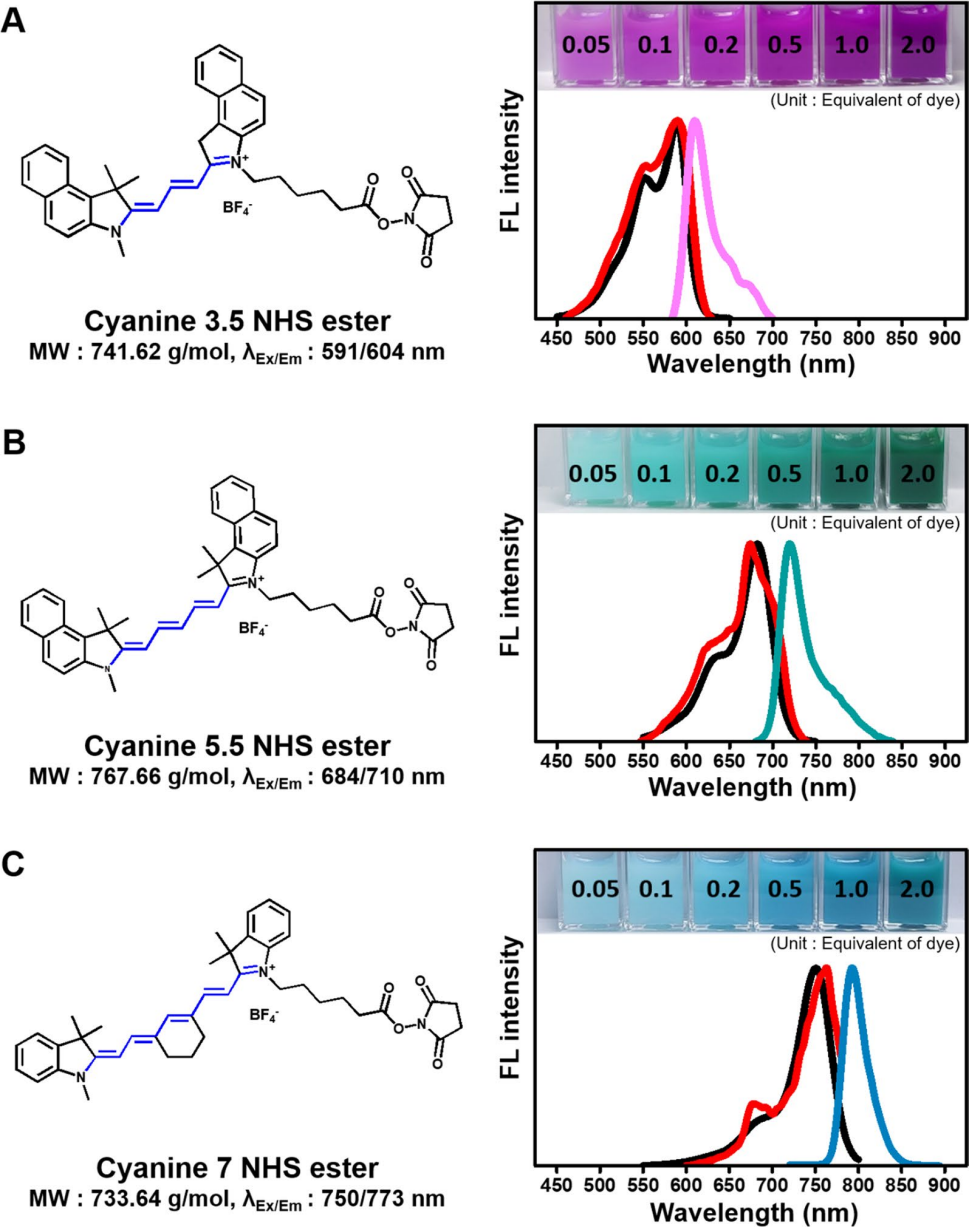
Structures and material properties of amine-reactive cyanine NHS ester dyes. The material properties of dye include molecular weights, fluorescence excitation/emission and experimental spectra of Cy3.5 (**A**), Cy5.5 (**B**), and Cy7 (**C**). The black spectrum indicates absorbance, the red spectrum indicates excitation scan, and the colored spectrum indicates emission scan. The numbers shown in the inset image represent the equivalents of dye. The molecular weights and fluorescence excitation/emission are referred from Lumiprobe

SNPs are favored because of their non-toxicity, stability, and excellent dispersibility in aqueous solutions [23, 24], rendering them suitable candidates for various biomedical applications, including imaging sensors. In this study, we explored the synthesis of fluorescent-dye-doped SNPs to optimize their imaging capabilities by meticulously examination of synthesis conditions. Three types of cyanine dyes, each with distinct excitation and emission properties, were used (Fig. 1).

**Fig. 2:**
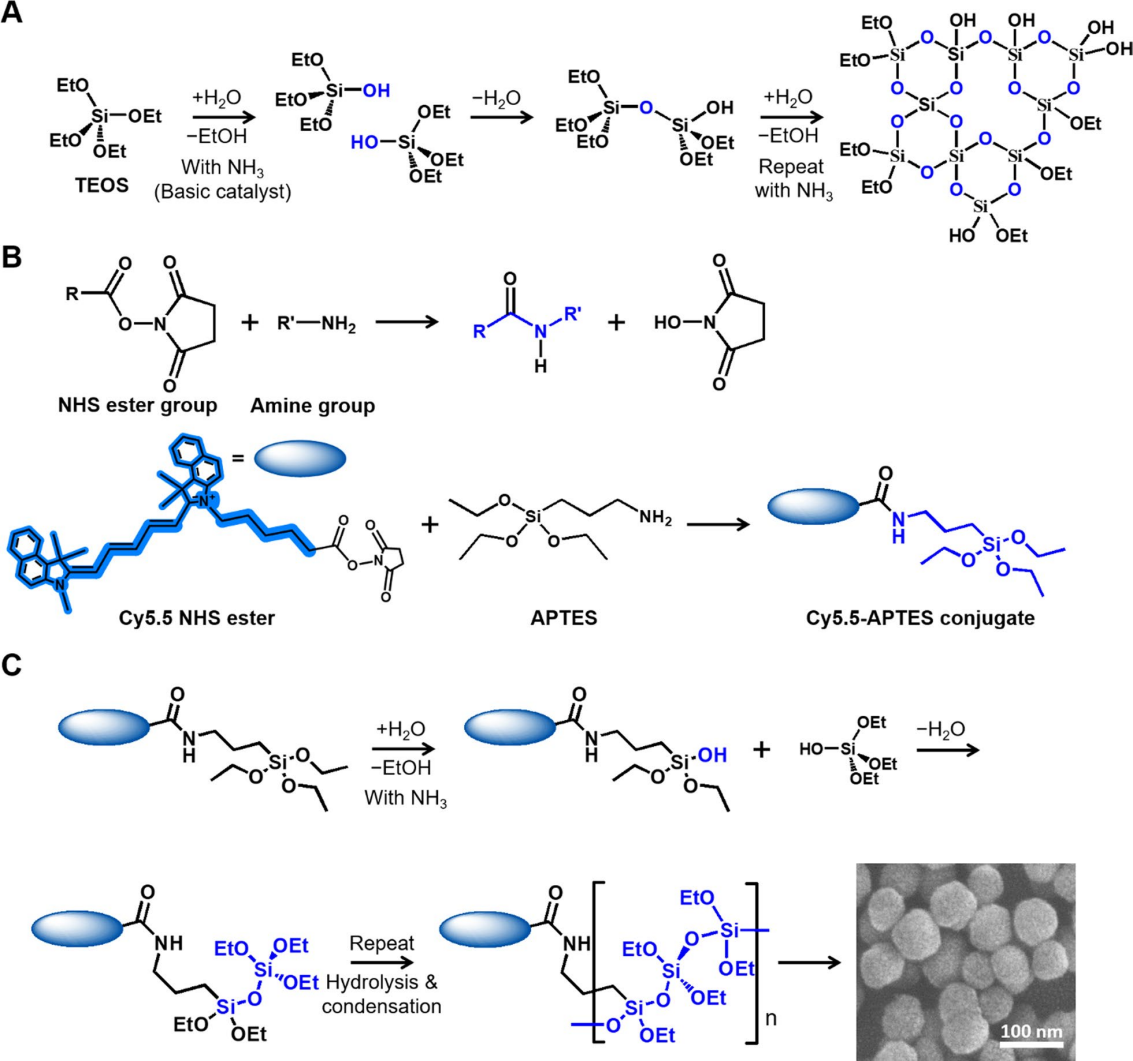
Synthetic reaction formula of silica nanoparticle (SNP)-based on Stöber method. **A** Ammonium catalyst based hydrolysis and condensation of silica. **B** Coupling reaction between the ester group on the cyanine dye and the amine group on APTES. **C** Fluorescent SNP formation with cyanine NHS ester dye. Scale bar = 100 nm

Fluorescent-dye-doped SNPs were synthesized using the Stöber method, in which the reaction proceeded in an aqueous solution (Fig. 2) [7]. Initially, APTES reacted with the NHS ester group of the dye to facilitate its binding to the particles, which was confirmed by mass spectrometry and NMR analyses. (Fig. S1). This was followed by condensation with tetraethyl orthosilicate (TEOS) to yield fluorescent SNPs with an average diameter of approximately 80 nm (Fig. S2, Table S1) [25]. Various synthesis conditions were explored, including the type of dye, dye-to-APTES ratio, and amount of dye added. The amount of dye was 1 equivalent, when dye-to-TEOS molar ratio was 1:5,000. Six types of particles added at 0.05, 0.1, 0.2, 0.5, 1.0, and 2.0 equivalents were synthesized, and their properties were compared. The synthesis ratios of the dyes, APTES, and TEOS are detailed in Tables 1 and S2.

**Table 1:** Molecular ratios used for the synthesis of fluorescent SNP

Group 1 (Dye:APTES = 1:50)			Group 2 (Dye:APTES = 1:100)		
Dye	APTES	TEOS	Dye	APTES	TEOS
0.05	**2.5**	5,000	0.05	**5**	5,000
0.1	**5**	5,000	0.1	**10**	5,000
0.2	**10**	5,000	0.2	**20**	5,000
0.5	**25**	5,000	0.5	**50**	5,000
1.0	**50**	5,000	1.0	**100**	5,000
2.0	**100**	5,000	2.0	**200**	5,000

Bold values indicate differences between the two groups

### Physical property analysis: morphological analysis

**Fig. 3:**
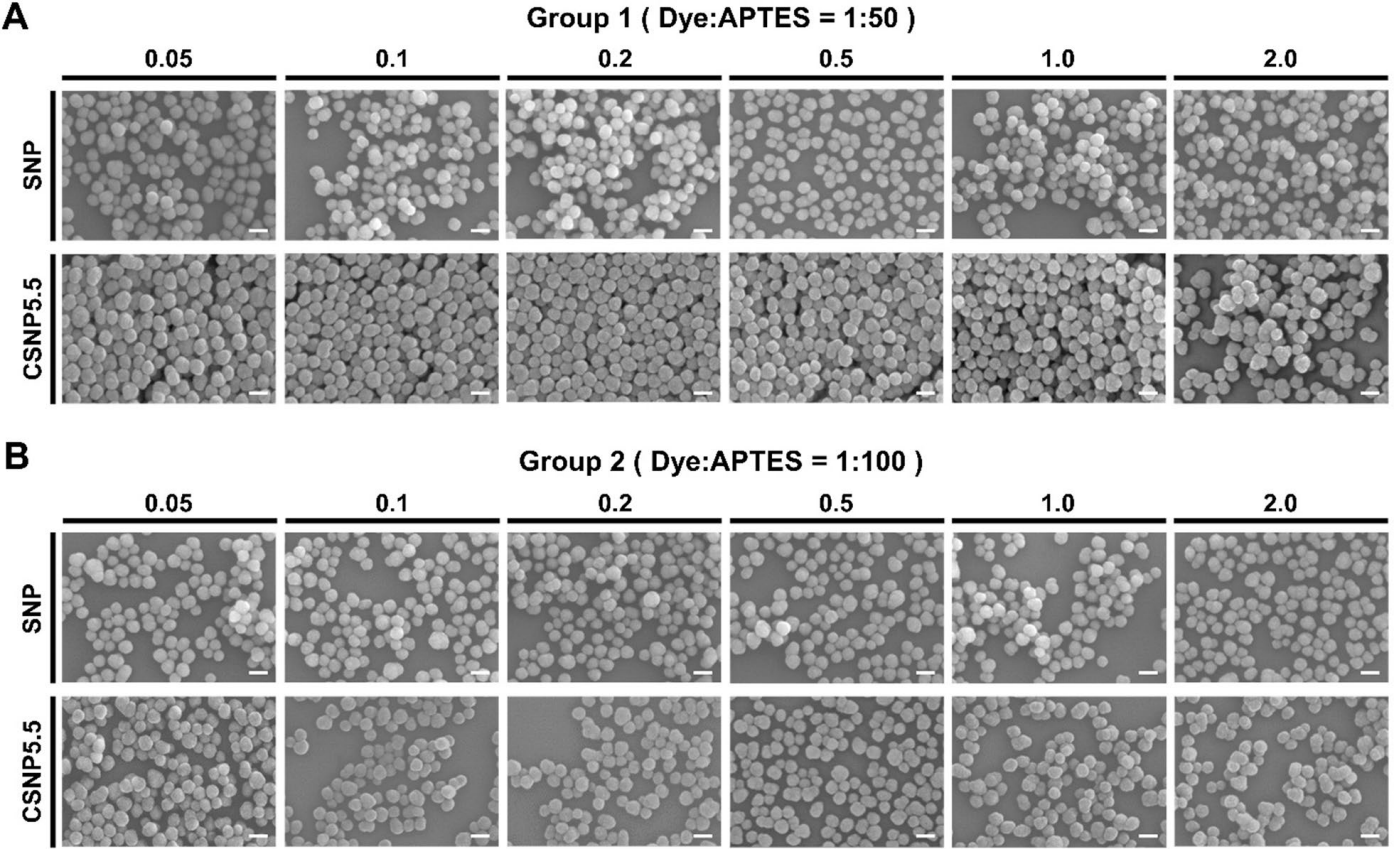
Morphological characteristics analyses. Representative scanning electron microscopy images of SNPs and CSNP5.5 synthesized at varying APTES ratios and equivalents. **A** Group synthesized with a 50-fold molar ratio excess of APTES to dye. **B** Group with a 100-fold. Within each group, nanoparticles were synthesized under various conditions depending on the equivalents. Scale bars = 100 nm

Regardless of the presence or type of dye being encapsulated, all the synthesized plain SNPs and CSNPs exhibited a spherical shape with a diameter of approximately 80 nm, indicating a uniform morphology (Figs. 3 and S3). Additionally, the nanoparticles demonstrated a narrow size distribution across all conditions, ensuring consistency in the particle size, as shown in Table S3. Despite changes in the APTES ratio and dye-equivalent conditions, no morphological differences were observed. Notably, the CSNPs exhibited good dispersibility in water, saline, and organic solvents, underscoring their versatility (Fig. S4). Figure S5 and Table S4 show the yields of the particles obtained through the synthesis.

### Physical property analysis: surface zeta potential

**Fig. 4:**
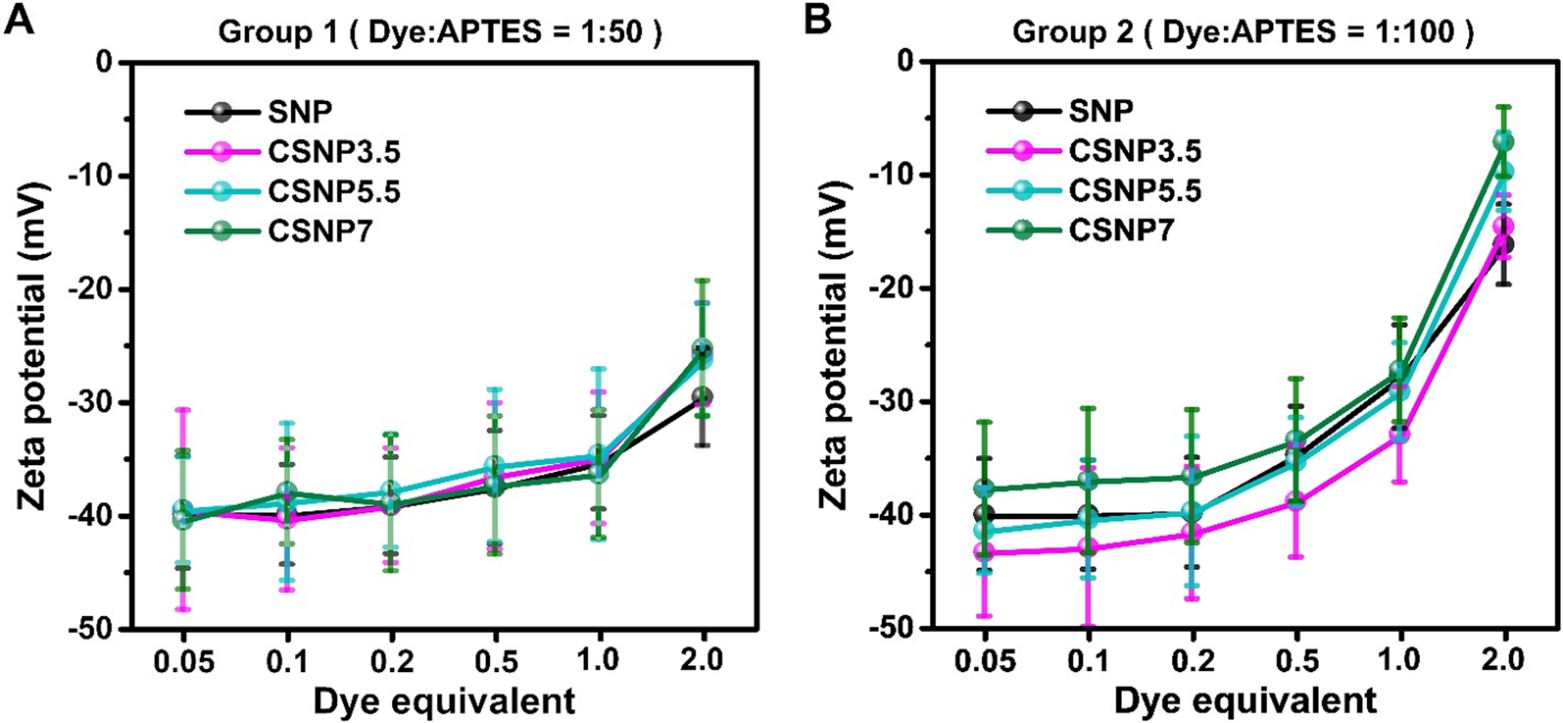
Surface charge of CSNPs. The zeta potentials of CSNPs synthesized under various APTES ratios and equivalents were measured using a DLS. **A** Group synthesized with a 50-fold molar ratio excess of APTES to dye. **B** Group with a 100-fold. The samples were suspended in distilled water with 2.5 mg/ml concentration and titrated to pH 7.4

Surface zeta potential analysis revealed a negative charge at a neutral pH for the synthesized nanoparticles (Fig. 4 and Table S5). Zeta potential values serve as indicators of dispersibility. All particles with an absolute zeta potential value greater than 30 mV (|ζ|≥ 30 mV) exhibit good dispersibility in aqueous solutions [26]. As the zeta potential approached zero, the dispersibility decreased. Both SNPs and CSNPs with a low equivalent amount of dye have zeta potential value of approximately − 40 mV. The values for both SNPs and CSNPs increased in proportion to the amount of dye and APTES, reaching approximately − 30 and − 10 mV for groups 1 and 2, respectively. Notably, no significant difference was observed in the zeta potential in the presence or absence of the dye. However, the increase was more pronounced in group 2 nanoparticles synthesized with higher APTES ratios. When comparing CSNP5.5 in each group, CSNP5.5 in group 1 showed an increase in zeta potential difference from − 39.6 to − 26.3 mV, a difference of 13.3, between 0.05 and 2 equivalents. In the case of group 2, the zeta potential increased by 31.8, from − 41.5 to − 9.7 mV. Consequently, in group 2, the dispersibility of particles with 2 equivalents decreased. This is generally attributed to the influence of positively charged APTES on the SNPs, which typically carry a negative charge [27].

### Dye encapsulation efficiency

**Fig. 5:**
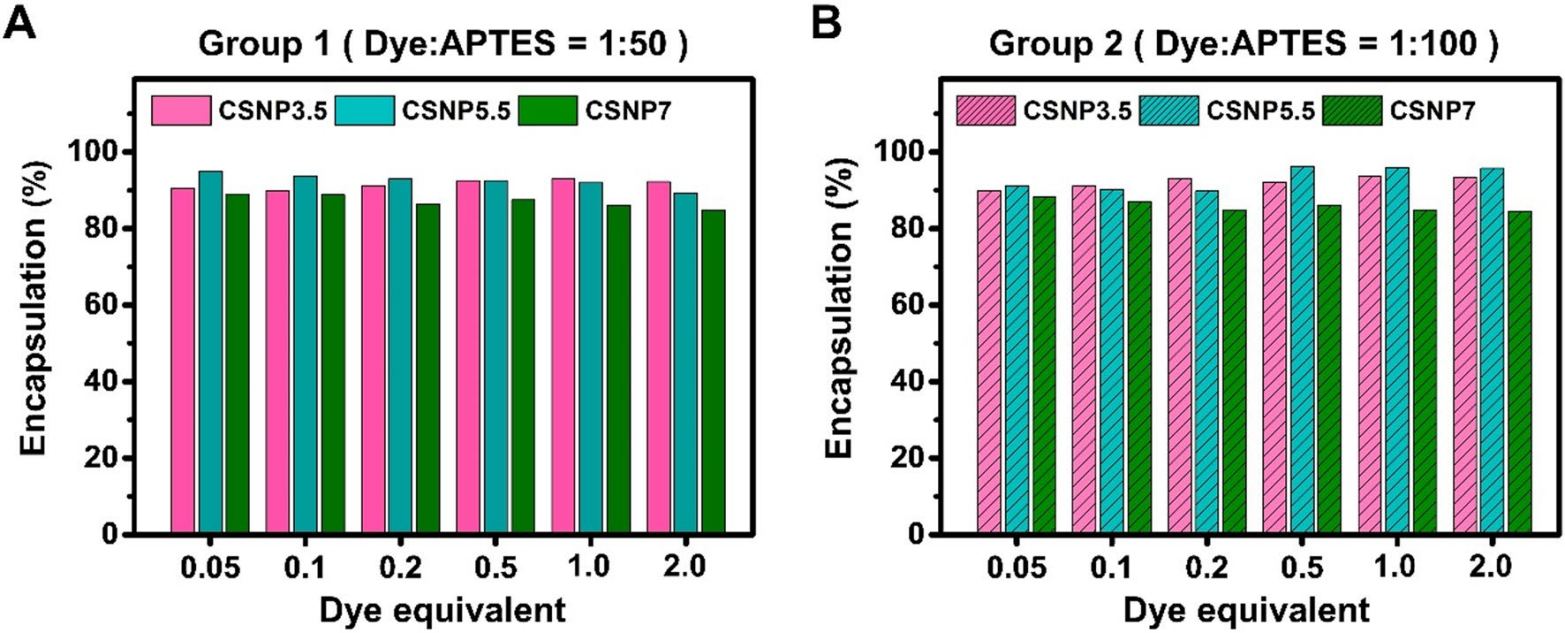
Encapsulation of cyanine dyes in CSNP synthesized under various APTES ratios and equivalents. **A** Group synthesized with a 50-fold molar ratio excess of APTES to dye. **B** Group with a 100-fold

During the synthesis, the encapsulation efficiency of the dye participating in the formation of CSNPs was measured. After synthesis, the supernatant was collected by centrifugation and washed twice, and the dye-APTES conjugate remaining in the supernatant was quantified. Encapsulation efficiency was calculated based on the difference between the amount of dye initially added to the reaction and the amount remaining in the supernatant after washing. Encapsulation efficiency was confirmed to be approximately 90% across all conditions (Figs. 5 and S6, and Table S6). This high efficiency underscores the effectiveness of the synthesis process for encapsulating the dye within the nanoparticles.

### Fluorescent property analysis: fluorescence emission spectra

**Fig. 6:**
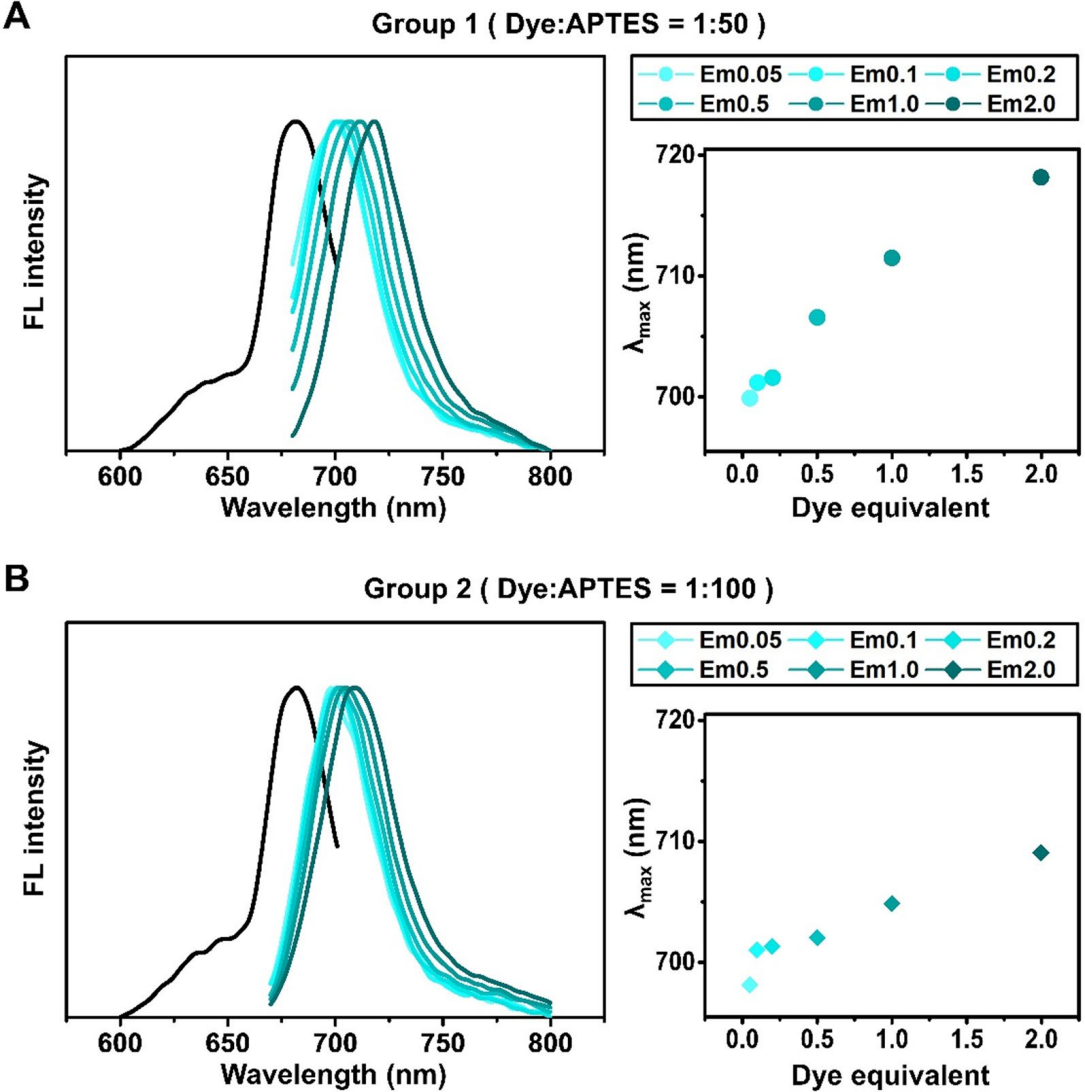
Emission spectra of CSNP5.5 synthesized under various APTES ratios and equivalents were measured using a fluorescence spectrophotometer. **A** Group synthesized with a 50-fold molar ratio excess of APTES to dye. **B** Group with a 100-fold. The black spectrum indicates excitation scan, and the cyanine series colored spectra indicate emission scan of each equivalent

After confirming the consistency of the size, morphology, and encapsulation efficiency of the CSNPs, we measured their excitation and emission spectra, focusing on CSNPs with Cy5.5 (CSNP5.5), in an ethanol aqueous solution (Fig. 6). The fluorescence emission spectra of the CSNPs exhibited a redshift, the extent of which depended on the amount of dye-APTES conjugate. The peak shift in group 2 was smaller (< 10 nm) than that in group 1 (< 18 nm). This redshift, indicative of the energy transfer between neighboring molecules and self-quenching [28], was more pronounced in nanoparticles synthesized with lower APTES conjugates. Although solvent interactions may also contribute to spectral shifts [29], the red shift observed in CSNPs is primarily influenced by intra-particle molecular interactions [30–32]. As the number of the dye molecules encapsulated within the nanoparticles increases, the distance between dye molecules gradually decreases. Consequently, as the distance decreases, a more active transfer of energy occurs between the molecules. During this intensified energy transfer process, energy loss occurs, resulting in a redshift [33]. In group 2, the smaller redshift may be due to the relatively greater distance between dye molecules, resulting from the higher APTES ratio compared to that in group 1. Similar trends were observed for CSNPs with Cy3.5 (CSNP3.5) and CSNPs with Cy7 (CSNP7), albeit with differences in the degree of shift (Fig. S7).

### Fluorescent property analysis: changes in fluorescent signal intensity depending on equivalent and concentration

**Fig. 7:**
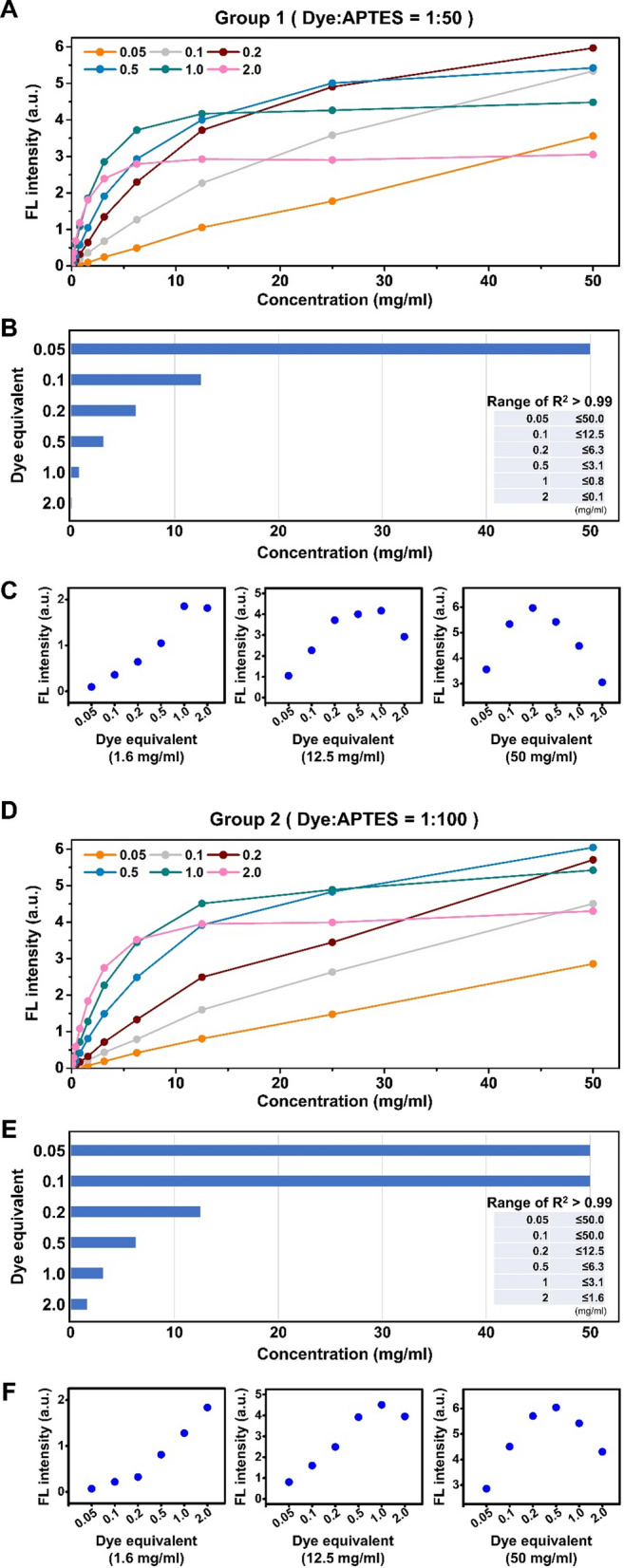
Comparison of fluorescent signal increase depending on the concentration of CSNP5.5 synthesized at varying APTES ratios and equivalents. **A**–**C** Group synthesized with a 50-fold molar ratio excess of APTES to dye. **D**–**F** Group with a 100-fold. **A**, **D** Increase of fluorescence signal with concentrations ranging from 0 to 50 mg/ml. **B**, **E** A range where the signal increases linearly (R^2^ > 0.99) in proportion to concentration. **F**, **D** Comparison of fluorescent signal efficiency at low, middle, and high concentrations. All signal intensities were obtained using a microplate reader

After establishing the spectra and peak wavelengths of the CSNPs, we measured the fluorescence signal intensity at concentrations ranging from 0 to 50 mg/ml (Fig. 7A and D). Based on our results, we identified linear ranges (R^2^ > 0.99) in which the fluorescent signal of each particle increased proportionally with concentration (Fig. 7B and E). In group 1, the linear signal increase range extended up to 50.0, 12.5, 6.3, 3.1, 0.8, and 0.1 mg/ml for 0.05, 0.1, 0.2, 0.5, 1, and 2 equivalent, respectively. In group 2, the linear signal increase range extended up to 50.0, 50.0, 12.5, 6.3, 3.1, and 1.6 mg/ml for 0.05, 0.1, 0.2, 0.5, 1, and 2 equivalent, respectively. The linear range of the signal intensity increase was wider for lower equivalents, and within each group, group 2 exhibited a wider range than group 1. Figure 7C and F show the signal intensity for each equivalent at low (1.6 mg/ml), middle (12.5 mg/ml), and high (50.0 mg/ml) particle concentrations. Although variations in the degree of signal intensity were observed depending on the dye used, a consistent trend was observed. At lower concentrations, CSNPs with higher equivalents exhibited stronger signals, whereas at higher concentrations, CSNPs with higher equivalents showed weaker signals, which was attributed to shorter distances between dye molecules within the SNPs leading to self-quenching. The results for CSNP3.5 and CSNP7 are shown in Figs. S8 and S9, respectively.

### In vitro and in vivo imaging application of CSNPs

**Fig. 8:**
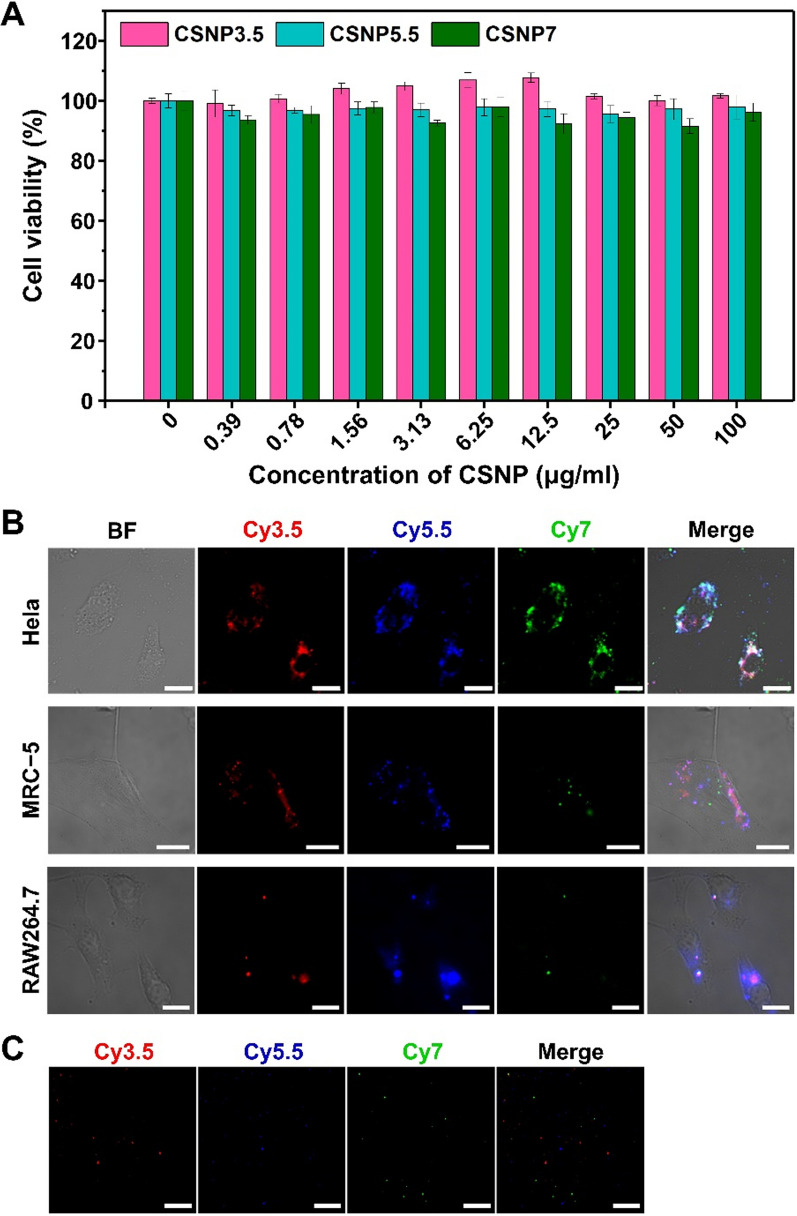
In vitro application of CSNPs. **A** Cytotoxicity of CSNPs to in vitro. The HeLa cells were incubated with CSNPs for 24 h and the cell viability were analyzed by MTS assay. **B** Wide field microscope images of CSNPs loaded to HeLa cell (scale bars = 30 μm), MRC-5 cell (20 μm), and Raw264.7 cell (10 μm). **C** Single particle microscope images of CSNPs after spin-coating (scale bars = 10 μm). The red, blue, and green colors indicate CSNP3.5, CSNP5.5, and CSNP7, respectively

Advances in molecular imaging have been crucial for disease diagnosis and treatment in biomedical research. The development of improved sensors for fluorescence imaging and multi-imaging systems is imperative for future advancements [14]. To demonstrate the applicability of CSNPs for in vitro and in vivo imaging, we performed experiments involving the particle treatment of cells and mice. When HeLa cells were treated with varying particle concentrations, no cell damage or death occurred after 24 h of incubation with the CSNPs (Fig. 8A).

Subsequently, fluorescence imaging of CSNP-treated cells using a wide-field microscope revealed the successful identification of signals within the cells, indicating effective uptake through the cell membrane (Fig. 8B). The excitation/emission of CSNP3.5 at 532/562 nm, CSNP5.5 at 664/675 nm, and CSNP7 at 785/800 nm were measured while taken up by live cells. Additionally, the particles were observed to colocalize with the cytoplasm in bright-field images, signifying successful uptake. These results indicate the low toxicity of CSNPs to cells and the ability to track the uptaken particles through long-term imaging or to compare and analyze treated particles simultaneously by adjusting the wavelength. Additionally, single-particle imaging of CSNPs was achieved after spin-coating (Fig. 8C), and by adjusting the wavelength, specific signals of the desired CSNPs could be obtained.

**Fig. 9:**
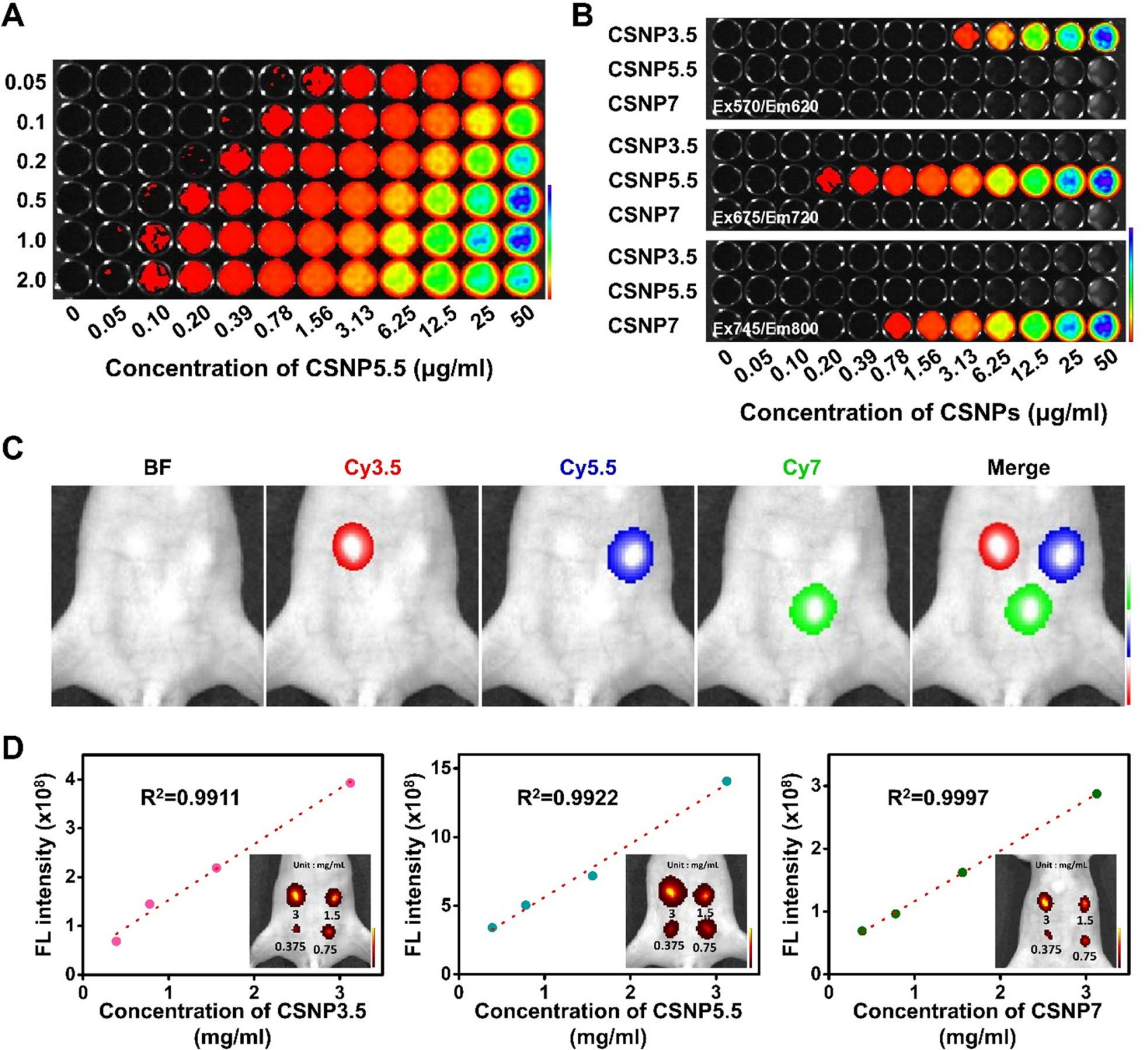
In vivo application of CSNPs. All imaging was conducted using an IVIS spectrum, with CSNPs being imaged at excitation/emission wavelengths of 570/620 nm, 675/720 nm, and 745/800 nm for CSNP3.5, CSNP5.5, and CSNP7, respectively. **A** Representative fluorescence image of CSNP5.5 in a black 96-well plate before injection into the mice. **B** Switchable imaging of CSNPs by their own wavelength. **C** In vivo fluorescence imaging of CSNPs. Red, blue, and green indicate CSNP3.5, CSNP5.5, and CSNP7, respectively. **D** Fluorescence intensities of CSNPs depending on concentration and their linearity

To demonstrate the feasibility of multi-imaging in both cells and animals, in vivo imaging was conducted using an optical imaging system (IVIS Spectrum, PerkinElmer, USA). The signal intensity of CSNP5.5 was influenced by both the amount of encapsulated dye conjugate and particle concentration (Fig. 9A). Notably, the imaging results shown in Fig. 9A for each equivalent correlate with the change in fluorescent signal values shown in Fig. 7. Furthermore, the selective imaging of CSNP3.5, CSNP5.5, and CSNP7 was achieved using their respective excitation/emission wavelengths (Fig. 9B). Subsequently, CSNPs dispersed in DPBS (Dulbecco’s phosphate buffered saline) were injected into different locations in the subcutaneous abdomen of mice, enabling imaging of the desired particles by adjusting the wavelengths as depicted in Fig. 9B (Fig. 9C). The fluorescent signal in the subcutaneous abdomen of mice increased linearly with particle concentration, suggesting that the linearity of fluorescence intensity at different concentrations was well maintained in vivo within subcutaneous tissue environments across the three different wavelengths (Fig. 9D). In addition, in vivo biodistribution imaging was performed to assess the biocompatibility and clearance of the synthesized nanoparticles, particularly CSNP7 (Fig. S10). While variations were noted depending on the injection dose, a significant signal decrease was observed within one day post-injection at the lower dose (3 mg/mouse), with the majority of the signal reduction confirmed three weeks later.

## Materials and methods

### Materials

Cyanine 3.5 N-hydroxysuccinimide ester (Cy3.5 NHS ester), cyanine 5.5 N-hydroxysuccinimide ester (Cy5.5 NHS ester), and cyanine 7 N-hydroxysuccinimide ester (Cy7 NHS ester) were purchased from Lumiprobe (USA). TEOS (98%) and APTES were purchased from Sigma-Aldrich (USA). Aqueous ammonia (28–30%) was obtained from Samchun Chemicals (Korea).

### Synthesis of cyanine dye-doped fluorescent silica nanoparticles

CSNPs were synthesized following a previously described method with slight modifications [7]. The synthesis was based on CSNP5.5, using Cy5.5 NHS ester dye. Initially, the Cy5.5-APTES conjugate was synthesized by stirring a mixture of Cy5.5 NHS ester and APTES in ethanol overnight at room temperature; the mixture contained 5 mg of Cy5.5 NHS ester dye and 152 µl of APTES per 10 ml of ethanol, resulting in a dye-to-APTES molar ratio of 1:100. CSNP5.5, with a diameter of less than 100 nm, was synthesized as follows: 2 ml of TEOS, 2.75 ml of the Cy5.5-APTES conjugate mixture, and 15.25 ml of ethanol were added to a 50 ml 1-neck round-bottom flask. A premixed solution of ammonium hydroxide (1.5 ml) and ethanol (8.5 ml) was then added to the reaction mixture and then stirred overnight at room temperature. As an imageable fluorescent nanoprobe, CSNP5.5 was synthesized with a TEOS:APTES:dye molar ratio of 5,000:100:1. Nanoprobes with varying amounts of encapsulated dye were produced by adjusting the quantity of the Cy5.5-APTES conjugate while maintaining a constant amount of TEOS. CSNPs were isolated and purified by centrifuging three times at 10,000 rpm, followed by dispersion and storage in ethanol at 4 °C until further use. During the creation of the conjugate mixture, omitting the addition of the dye and proceeding with the synthesis resulted in the fabrication of SNPs (Table S2).

### Physical property analysis

Morphological analyses of the CSNPs were performed using field-emission scanning electron microscopy (Merlin Compact, Carl Zeiss, Germany). The particle size distribution and zeta potential were analyzed using a Zetasizer instrument (Nano ZS, Malvern Instruments Ltd, UK).

### Dye encapsulation efficiency

The dye encapsulation efficiency was determined by measuring the amount of residual dye in the supernatant. After synthesis, the particles were isolated and purified by centrifuging at 10,000 rpm for 20 min. During this step, the supernatant was collected separately, and the absorbance of the dye-APTES conjugate remaining in the solution was measured using a UV–Vis spectrometer (UV-2600, Shimadzu, Japan). The dye encapsulation efficiency was calculated based on the difference between the initial and remaining amounts of dye in the supernatant.

### Fluorescent property analysis

The fluorescence excitation and emission spectra were recorded using a fluorescence spectrophotometer (FS2, Scinco, Korea). The samples were then dispersed in ethanol. CSNPs were dispersed in ethanol and prepared at concentrations ranging from 0 to 50 mg/ml by serial dilution to compare fluorescent signal intensity by concentration. Subsequently, 100 µl of each sample was added to each well of a black 96-well plate, which was measured using a microplate reader (SpectraMax M4, Molecular Devices, USA).

### In vitro application of CSNPs

Cytotoxicity assays of the CSNPs were performed using MTS solution (Ez-cytoX, DoGenBio, Korea). HeLa (human cervical cancer) cells were seeded at a density of 1 × 10^4^ cells/well in 100 µl of Dulbecco;s Modified Eagle Medium (DMEM; Gibco, USA) in a 96-well plate. The following day, each CSNP was serially diluted starting from a maximum concentration of 100 µg/ml and added to the wells. After 24 h of incubation with CSNPs, cell viability was measured at 450 nm using a microplate spectrophotometer (xMark^™^, BioRAD, USA). Data shown are the averages of four independent experiments.

MRC-5 (human lung fibroblast) cells and Raw264.7 (mouse macrophage) cells were maintained as monolayers in a humidified incubator (5% CO_2_) in Eagle’s Minimum Essential Medium (MEM; Gibco) and DMEM supplemented with 10% (v/v) fetal bovine serum (Gibco) and 100 IU/ml penicillin–streptomycin solution (Gibco). Before treatment with CSNPs, the cells were seeded at a density of 1 × 10^4^ cells/well using 8-well chamber slides (μ-slide, ibidi, Germany) for 12 h. The cells were then treated with 1 μg/ml of the CSNP mixture in Opti-MEM media (Gibco) for 1 h. After CSNP treatment, the cells were fixed with 4% formaldehyde, washed three times with DBPS, and mounted with mounting solution (Dako, USA) for fluorescence imaging.

The CSNP-treated cells on the μ-slide were placed on an oil immersion-type objective lens (UPlanSApo 60X, Olympus, Japan) in an inverted microscope (IX71, Olympus). Emission images were captured using an electron-multiplying charge-coupled device (iXon3, DU-888D-C00-#EX, Andor, UK). For the excitation of CSNP3.5, CSNP5.5, and CSNP7, CW diode-pumped lasers with wavelengths of 532 (0532-04-01 Samba^™^, Cobolt, Sweden), 660 (Flamenco^™^, Cobolt), and 785 (SN0852290, Lumics, Germany) were used, respectively. For each laser, dichroic beam-splitters (LPD01-532R, FF665-Di02, and NFD01-785; Semrock, USA) were used to block the excitation wavelength. Specific optical filters were employed to block the excitation wavelength and capture emissions from CSNPs as follows: a long-pass filter (LP03-532RE, Semrock) and a bandpass filter (FF01-562-40/40, Semrock) for CSNP3.5, a long-pass filter (LP02-664RU-25, Semrock) and a bandpass filter (FF02-675/67-25, Semrock) for CSNP5.5, and a long-pass filter (LPD01-785RU, Semrock) and a bandpass filter (FF01-800/12, Semrock) for CSNP7, respectively. All cells were confirmed to be mycoplasma-free using a mycoplasma detection kit (InvivoGen, USA).

The CSNPs were spin-coated onto a piranha-etched cover glass to obtain single-particle level images using a homemade wide-field nanospectroscopic imaging system [34].

### In vivo application of CSNPs

In vivo imaging was performed using an optical imaging system (IVIS Spectrum) with 1 equivalent of CSNPs and an APTES ratio of 1:100. Representative fluorescence images of CSNPs were obtained by preparing samples with several concentrations on a blackplate, ranging from the highest concentration of 50 to 0.05 mg/ml through serial dilution, and adding 100 µl of sample to each well. Imaging was conducted at specific excitation/emission wavelengths of 570/620, 675/720, and 745/800 nm for CSNP3.5, CSNP5.5, and CSNP7, respectively. CSNPs were subcutaneously injected into the abdomen of BALB/c nude mice at different locations at varying concentrations (0.375, 0.75, 1.5, and 3 mg/ml), followed by imaging to confirm the linearity of the signal.

## Conclusions

Through our comprehensive investigation of silica nanoparticles incorporating the three types of dyes, we garnered valuable insights into their physical and fluorescent properties. Our findings confirmed the consistent size and shape of the silica nanoparticles across various synthesis conditions, while highlighting notable changes in the surface charge and fluorescence characteristics contingent upon the amount of encapsulated dye-APTES. These observations underscore the significance of precise control over synthesis parameters for tailoring nanoparticle properties for specific imaging applications. In addition, our in vitro and in vivo experiments confirmed the versatility and utility of these fluorescent silica nanoparticles. We observed a linear increase in signal intensity with particle concentration, even in animal skin, indicating a robust performance in imaging applications. These results provide practical guidance for experimental design, facilitating informed decisions regarding dye selection, encapsulation quantity, and particle concentration, tailored to specific imaging objectives and durations. Continuous research on fluorescent nanoparticles holds promise for the further advancement of in vivo imaging capabilities. In addition to imaging, these nanoparticles present opportunities for use as versatile monitoring tools in drug delivery and nanomedicine research. By leveraging their unique properties, future endeavors in this realm aim to enhance the performance and broaden the scope of applications, ultimately advancing biomedical research and clinical practices.

### Supplementary Information

Supplementary Material 1.

## Data Availability

The datasets in the current study are included in the published article or available from the corresponding author on reasonable request.

## Abbreviations

CSNP: Cyanine dye-doped fluorescent silica nanoparticle
SNP: Plain silica nanoparticle
Cy3.5: Cyanine 3.5
Cy5.5: Cyanine 5.5
Cy7: Cyanine 7
NHS ester: N-hydroxysuccinimide ester
APTES: (3-Aminopropyl)triethoxysilane
TEOS: Tetraethyl orthosilicate
CSNP5.5: CSNP with Cy5.5
CSNP3.5: CSNP with Cy3.5
CSNP7: CSNP with Cy7

## References

[1] Xiao D, Qi H, Teng Y, Pierre D, Kutoka PT, Liu D (2021). Advances and challenges of fluorescent nanomaterials for synthesis and biomedical applications. Nanoscale Res Lett.

[2] Anselmo AC, Mitragotri S (2021). Nanoparticles in the clinic: an update post COVID-19 vaccines. Bioeng Transl Med.

[3] Ma D, Kell AJ, Tan S, Jakubek ZJ, Simard B (2009). Photophysical properties of dye-doped silica nanoparticles bearing different types of dye-silica interactions. J Phys Chem C.

[4] Janjua TI, Cao Y, Yu C, Popat A (2021). Clinical translation of silica nanoparticles. Nat Rev Mater.

[5] Kharlamov AN, Tyurnina AE, Veselova VS, Kovtun OP, Shur VY, Gabinsky JL (2015). Silica–gold nanoparticles for atheroprotective management of plaques: results of the NANOM-FIM trial. Nanoscale.

[6] Kharlamov AN, Feinstein JA, Cramer JA, Boothroyd JA, Shishkina EV, Shur V (2017). Plasmonic photothermal therapy of atherosclerosis with nanoparticles: long-term outcomes and safety in NANOM-FIM trial. Future Cardiol.

[7] Rossi LM, Shi L, Quina FH, Rosenzweig Z (2005). Stöber synthesis of monodispersed luminescent silica nanoparticles for bioanalytical assays. Langmuir.

[8] Yang SA, Choi S, Jeon SM, Yu J (2018). Silica nanoparticle stability in biological media revisited. Sci Rep.

[9] Mulvaney P, Liz-Marzán LM, Giersig M, Ung T (2000). Silica encapsulation of quantum dots and metal clusters. J Mater Chem.

[10] Cao A, Ye Z, Cai Z, Dong E, Yang X, Liu G, Deng X, Wang Y, Yang ST, Wang H, Mu M, Liu Y (2010). A facile method to encapsulate proteins in silica nanoparticles encapsulated green. Angew Chem Int Ed.

[11] Yang Y, Zhang M, Song H, Yu C (2020). Silica-based nanoparticles for biomedical applications: from nanocarriers to biomodulators. Acc Chem Res.

[12] Son T, Cho YJ, Lee H, Cho MY, Goh B, Kim HM, Phan TNH, Cho SH, Park YJ, Park HS, Hong KS (2022). Monitoring in vivo behavior of size-dependent fluorescent particles as a model fine dust. J Nanobiotechnol.

[13] Manzano M, Vallet-Regí M (2020). Mesoporous silica nanoparticles for drug delivery. Adv Funct Mater.

[14] Refaat A, Yap ML, Pietersz G, Walsh APG, Zeller J, Del Rosal B, Wang X, Peter K (2022). In vivo fluorescence imaging: success in preclinical imaging paves the way for clinical applications. J Nanobiotechnol.

[15] Bringley JF, Penner TL, Wang R, Harder JF, Harrison WJ, Buonemani L (2008). Silica nanoparticles encapsulating near-infrared emissive cyanine dyes. J Coll Interface Sci.

[16] Hao Q, Qiu T, Chu PK (2012). Surfaced-enhanced cellular fluorescence imaging. Prog Surf Sci.

[17] Zelmer A, Ward TH (2013). Noninvasive fluorescence imaging of small animals. J Microsc.

[18] Sypabekova M, Hagemann A, Rho D, Kim S (2023). Review: 3-Aminopropyltriethoxysilane (APTES) deposition methods on oxide surfaces in solution and vapor phases for biosensing applications. Biosensors.

[19] Krusemark CJ, Frey BL, Smith LM, Belshaw PJ (2011). Complete chemical modification of amine and acid functional groups of peptides and small proteins. Methods Mol Biol.

[20] Rizvi SAA, Saleh AM (2018). Applications of nanoparticle systems in drug delivery technology. Saudi Pharm J.

[21] Karan S, Cho MY, Lee H, Kim HM, Park HS, Han EH, Sessler JL, Hong KS (2023). Hypoxia-directed and self-immolative theranostic agent: imagingand treatment of cancer and bacterial infections. J Med Chem.

[22] Wang W, Nallathamby PD, Foster CM, Morrell-Falvey JL, Mortensen NP, Doktycz MJ, Gu B, Retterer ST (2013). Volume labeling with Alexa Fluor dyes and surface functionalization of highly sensitive fluorescent silica(SiO2) nanoparticles. Nanoscale.

[23] Wang K, He X, Yang X, Shi H (2013). Functionalized silica nanoparticles: a platform for fluorescence imaging at the cell and small animal levels. Acc Chem Res.

[24] Sun D, Kang S, Liu C, Lu Q, Cui L, Hu B (2016). Effect of zeta potential and particle size on the stability of SiO_2_ nanospheres as carrier for ultrasound imaging contrast agents. Int J Electrochem Sci.

[25] Hamidon TS, Hussin MH (2020). Susceptibility of hybrid sol-gel (TEOS-APTES) doped with caffeine as potent corrosion protective coatings for mild steel in 3.5 wt.% NaCl. Prog Org Coat.

[26] Bukara K, Schueller L, Rosier J, Martens MA, Daems T, Verheyden L, Eelen S, Speybroeck MV, Libanati C, Martens JA, Mooter GVD, Frérart F, Jolling K, Gieter MD, Bugarski B, Kiekens F (2016). Ordered mesoporous silica to enhance the bioavailability of poorly water-soluble drugs: proof of concept in man. Eur J Pharm Biopharm.

[27] Ngouangna EN, Manan MA, Oseh JO, Norddin MNAM, Agi A, Gbadamosi AO (2020). Influence of (3–Aminopropyl) triethoxysilane on silica nanoparticle for enhanced oil recovery. J Mol Liq.

[28] Smith GC, Sinski JF (1999). The red-shift cascade: investigations into the concentration-dependent wavelength shifts in three-dimensional fluorescence spectra of petroleum samples. Appl Spectrosc.

[29] Fodor L, Lendvay G, Horváth A (2007). Solvent dependence of absorption and emission spectra of Ru(bpy)2(CN)2: experiment and explanation based on electronic structure theory. J Phys Chem A.

[30] Rampazzo E, Bonacchi S, Montalti M, Prodi L, Zaccheroni N (2007). Self-organizing core−shell nanostructures spontaneous accumulation of dye in the core of doped silica nanoparticles. J Am Chem Soc.

[31] Imhof A, Megens M, Engelberts JJ, de Lang DTN, Sprik R, Vos WL (1999). Spectroscopy of fluorescein (FITC) dyed colloidal silica spheres. J Phys Chem B.

[32] Lakowicz JR, Lakowicz JR (2006). Instrumentation for fluorescence spectroscopy. Principles of fluorescence spectroscopy.

[33] Malfatti L, Kidchob T, Aiello D, Aiello R, Testa F, Innocenzi P (2008). Aggregation states of rhodamine 6G in mesostructured silica films. J Phys Chem C.

[34] Kim J, Park HS, Ahn Y, Cho YJ, Shin HH, Hong KS, Nam SH (2023). Universal emission characteristics of upconverting nanoparticles revealed by single-particle spectroscopy. ACS Nano.

