# Supplementary material for: Advancing fluorescence imaging: enhanced control of cyanine dye-doped silica nanoparticles
Source: J Nanobiotechnology. 2024 Jun 19;22:347. doi: 10.1186/s12951-024-02638-7 (PMC11188253; doi:10.1186/s12951-024-02638-7)
Supplement: Supplementary file 1 — Supplementary Material 1. [file 12951_2024_2638_MOESM1_ESM.docx]

Supplementary Information for

**Advancing fluorescence imaging: Enhanced control of cyanine dye-doped silica nanoparticles**

Taewoong Son^1,2^, Minseo Kim^3,4^, Minsuk Choi^3^, Sang Hwan Nam^3^, Ara Yoo^1^, Hyunseung Lee^1^, Eun Hee Han^1,5^, Kwan Soo Hong^1,2,6,*^ and Hye Sun Park^1,*^

^1^Biopharmaceutical Research Center, Korea Basic Science Institute, Cheongju 28119, Republic of Korea.

^2^Graduate School of Analytical Science and Technology, Chungnam National University, Daejeon 34134, Republic of Korea.

^3^Laboratory of Nanophotonics & Nanospectroscopic Imaging, Korea Research Institute of Chemical Technology, Daejeon 34114, Republic of Korea.

^4^Department of Chemistry, Sungkyunkwan University, Suwon 16419, Republic of Korea.

^5^Korea University of Science and Technology, Daejeon 34113, Republic of Korea.

^6^Department of Chemistry, Chung-Ang University, Seoul 06974, Republic of Korea.

Keywords : Cyanine N-hydroxysuccinimide ester, Silica nanoparticle, Characterization, Fluorescence *in vitro* and *in vivo* image, Imaging optimization.


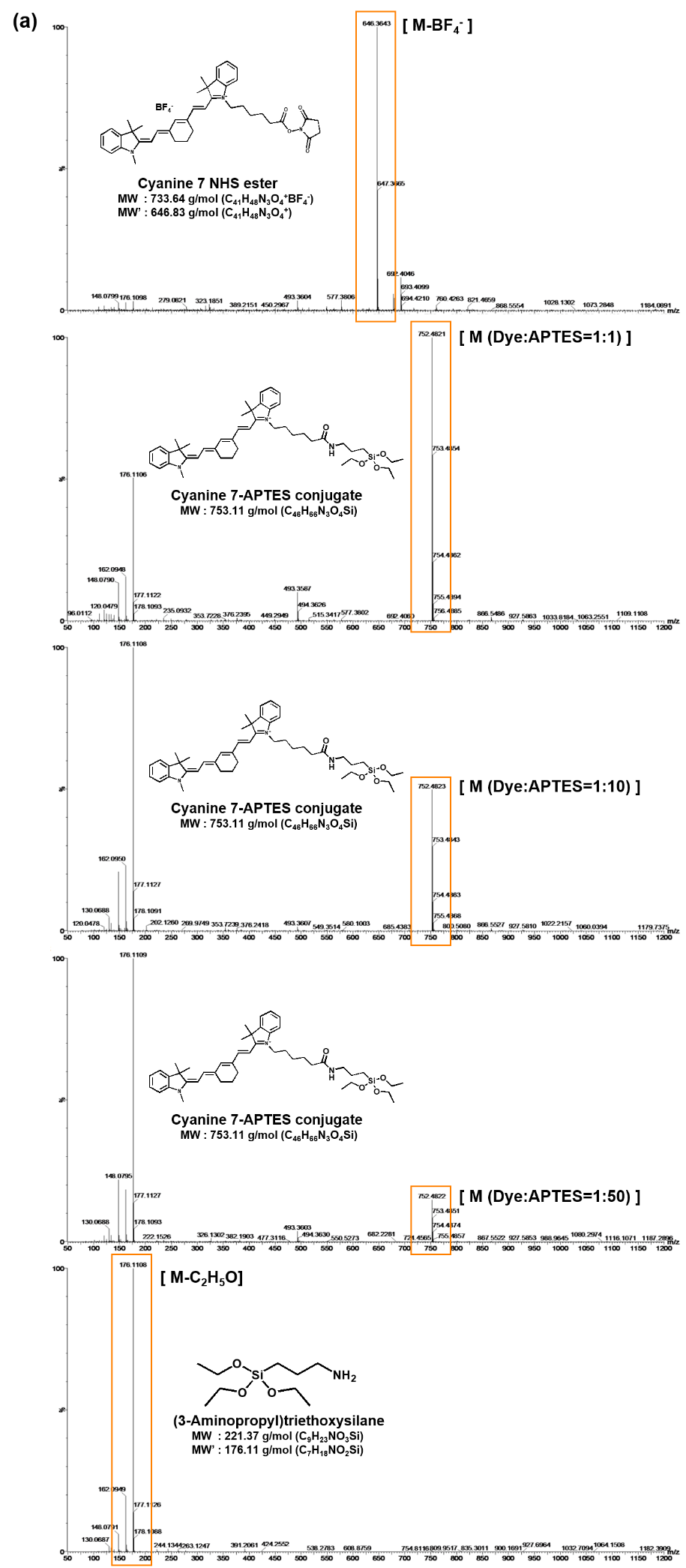


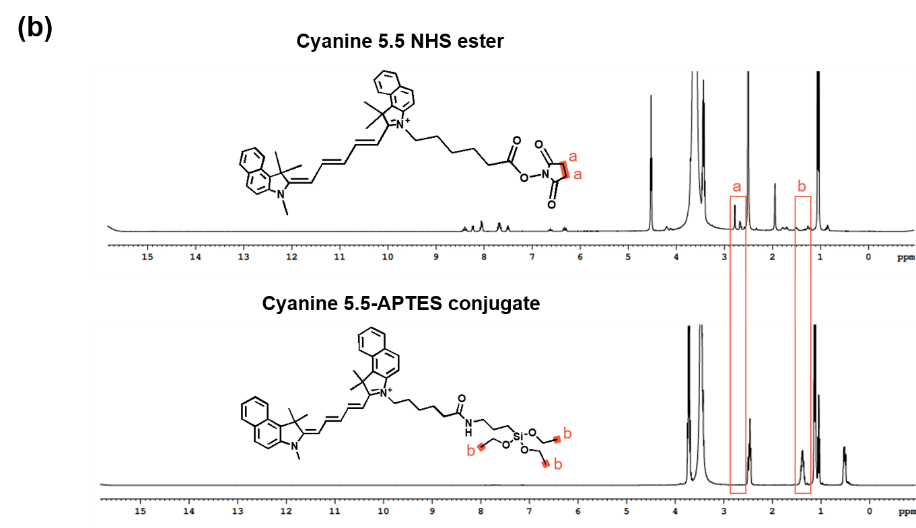


**Figure S1.** Confirmation of dye-APTES(3-Aminopropyl)triethoxysilane) conjugation. (a) Mass spectra of cyanine 7 NHS ester, cyanine 7-APTES conjugates, and APTES, obtained using ESI-TOF MS (SYNAPT G2, Waters, UK). (b) ^1^H-NMR of cyanine 5.5 NHS ester and cyanine 5.5-APTES conjugate in DMSO-d_6_, obtained using 400MHz NMR (Avance III 400, Bruker, USA) [1].

[1] Shirini F, Jolodar OG, Seddighi M, Borujeni HT. Preparation, characterization and application of succinimidinium hydrogensulfate ([H-Suc]HSO4) as an efficient ionic liquid catalyst for the N-Boc protection of amines. RSC Adv. 2015;5:19790
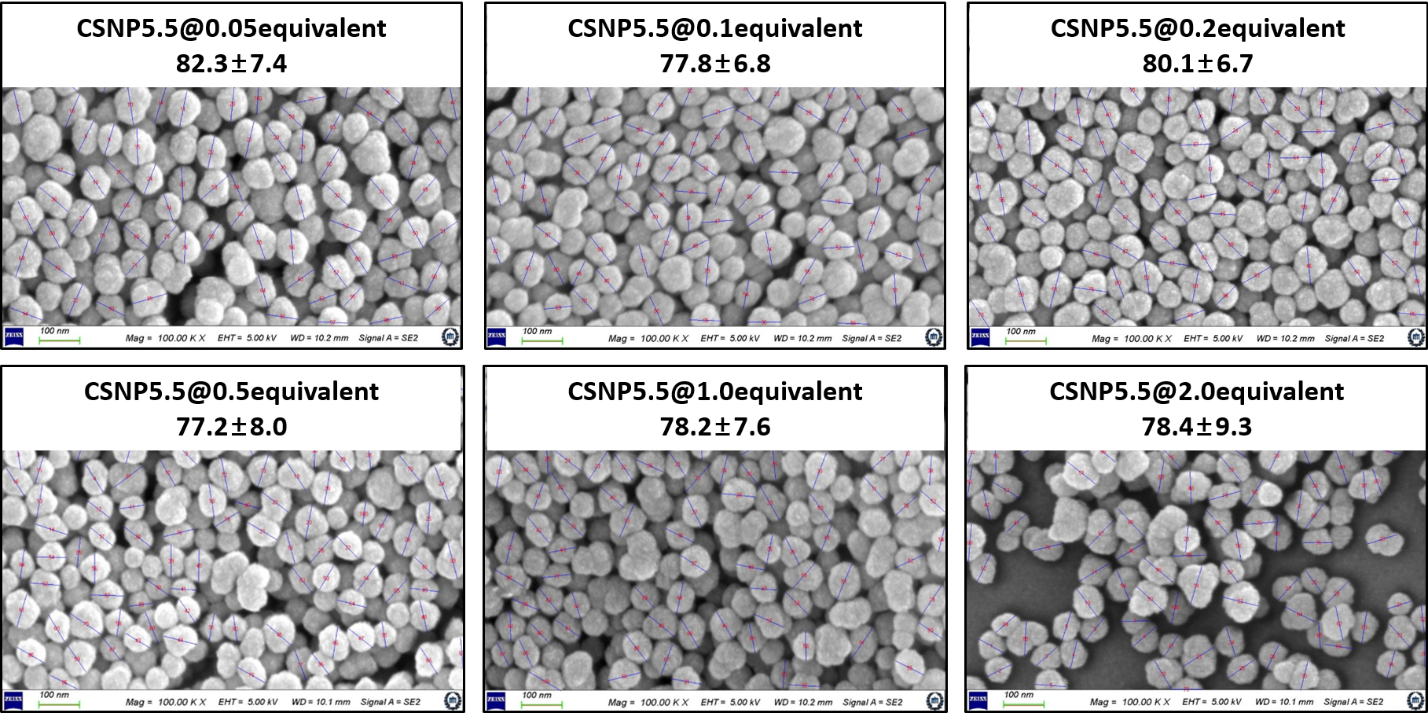


**Figure S2.** Size measurement of CSNP5.5 in group 1 using Nano Measurer software.


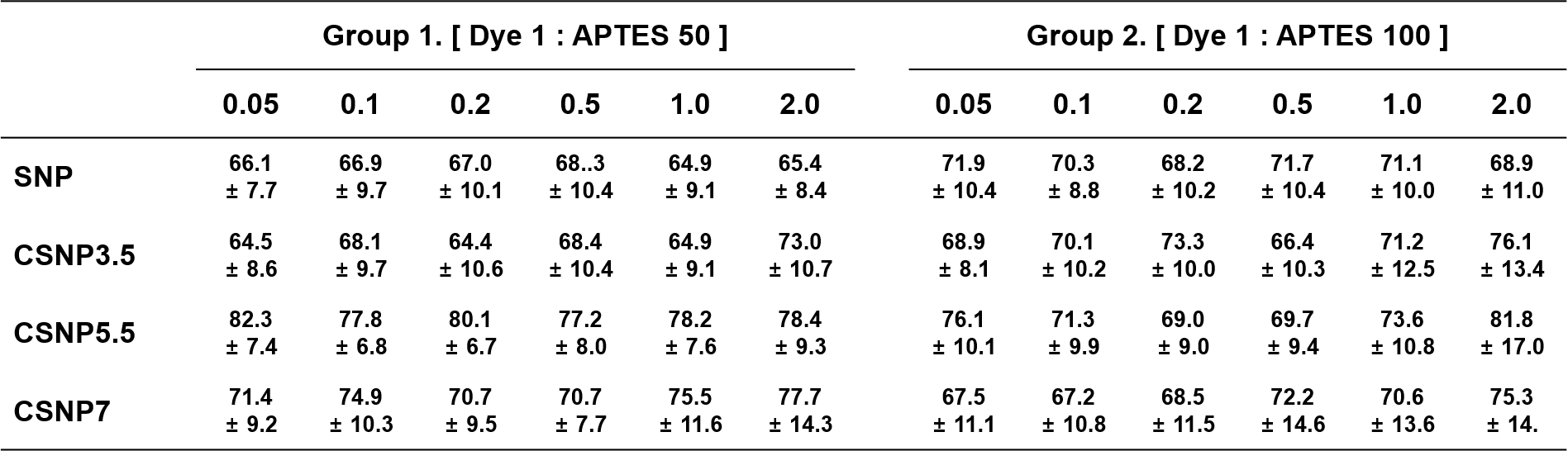


**Table S1.** Size measurement of SNP and CSNPs. The particle diameters were measured by Nano Measurer software using SEM images (n = 100).


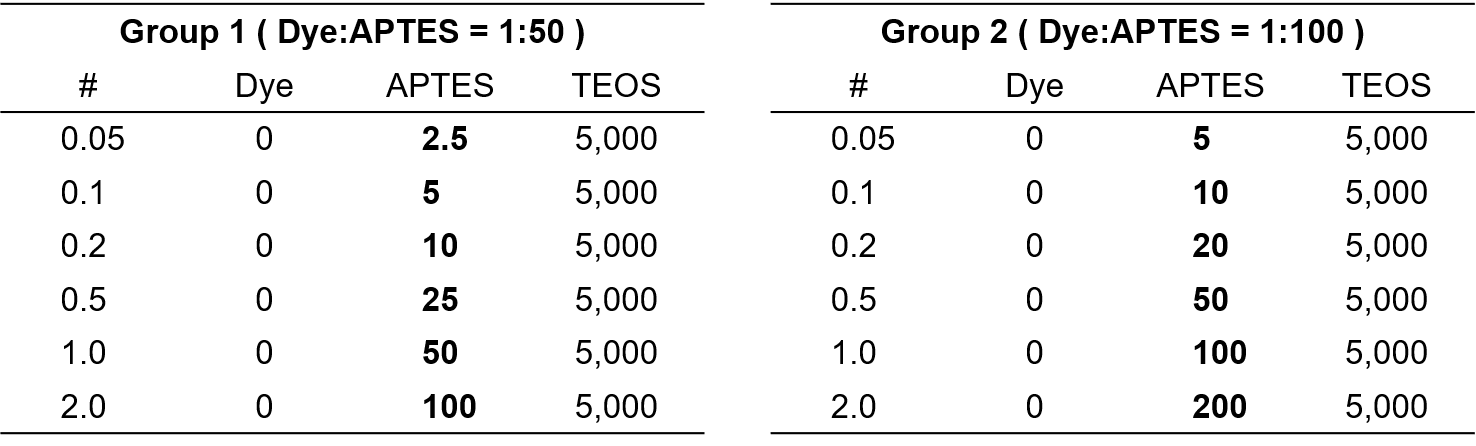


**Table S2.** Molecular ratios used for synthesis of plain silica nanoparticle.


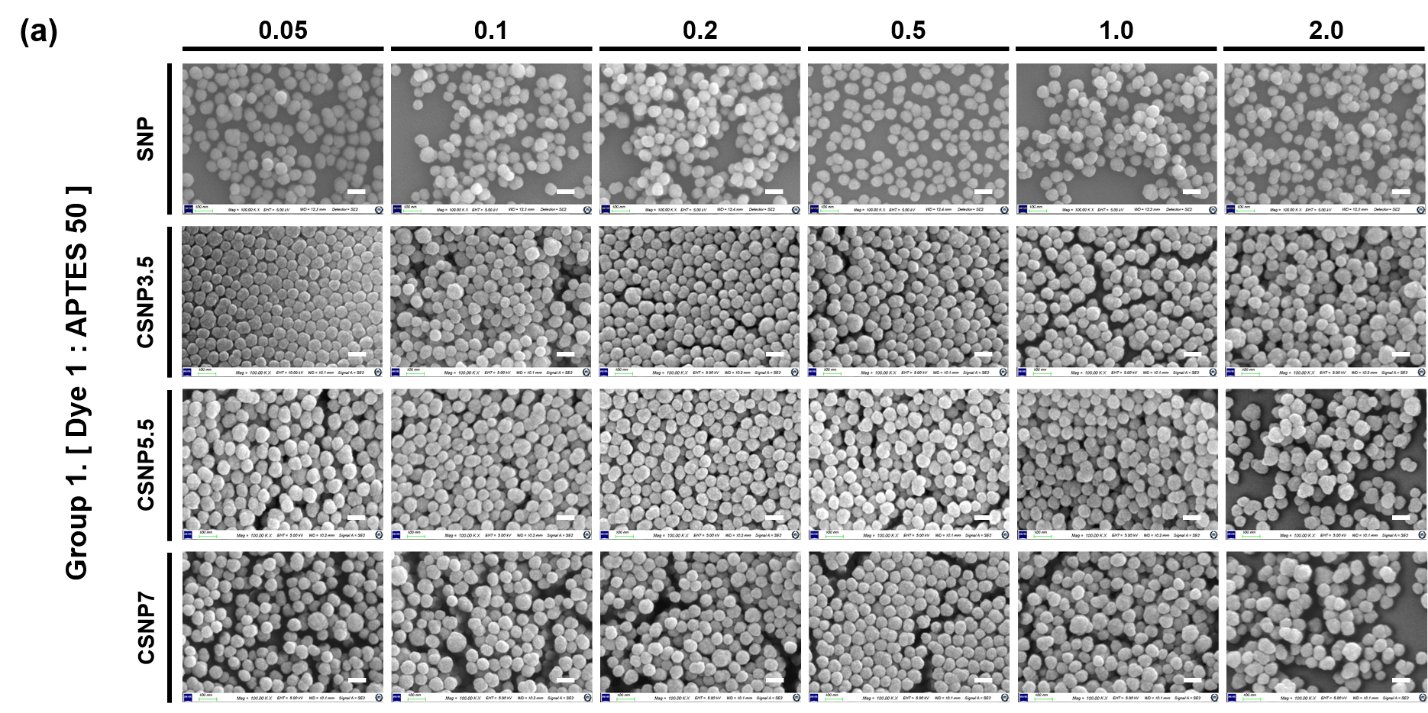


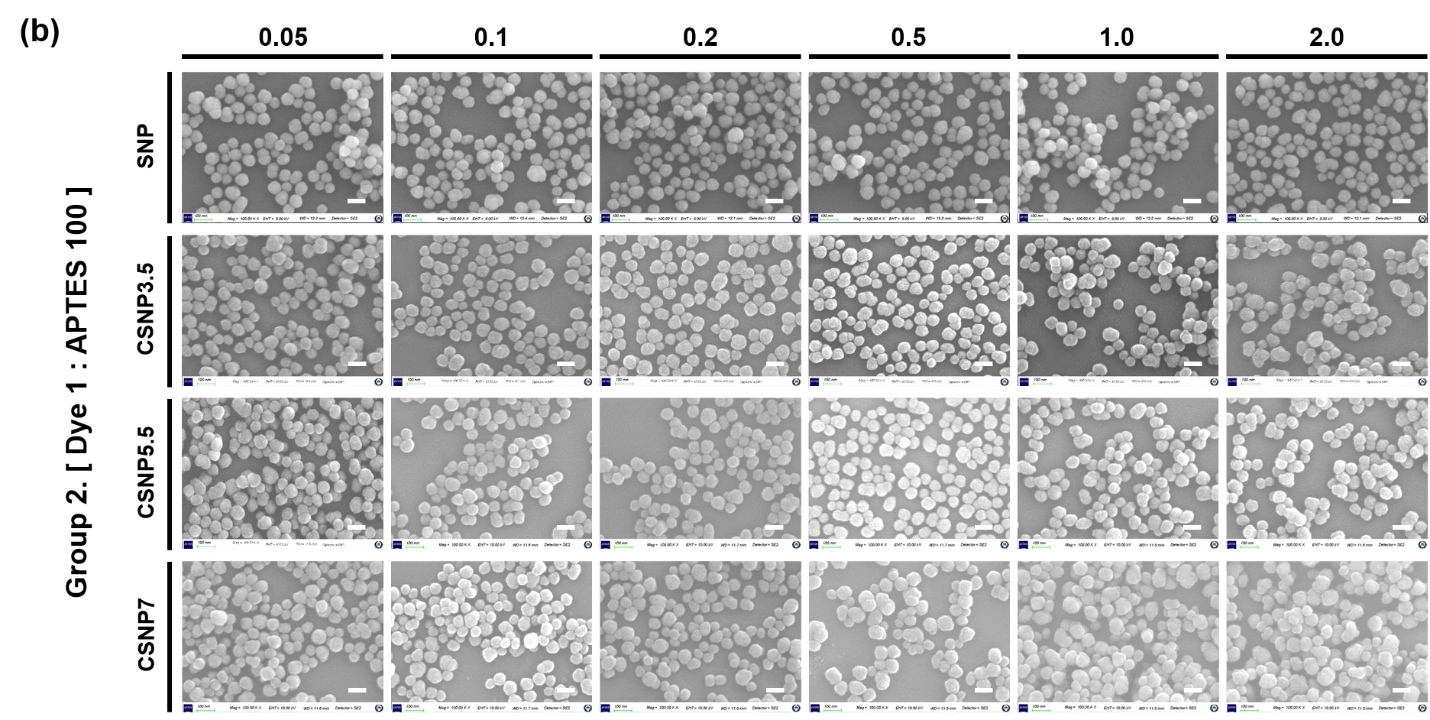


**Figure S3.** Morphological characteristics analyses. Scanning electron microscope (SEM) images of SNP and CSNPs synthesized under various APTES ratios and equivalents. (a) Group synthesized with a 50-fold molar ratio excess of APTES to dye. (b) Group with a 100-fold. Within each group, nanoparticles were synthesized under various conditions depending on the equivalents. Scale bars = 100 nm


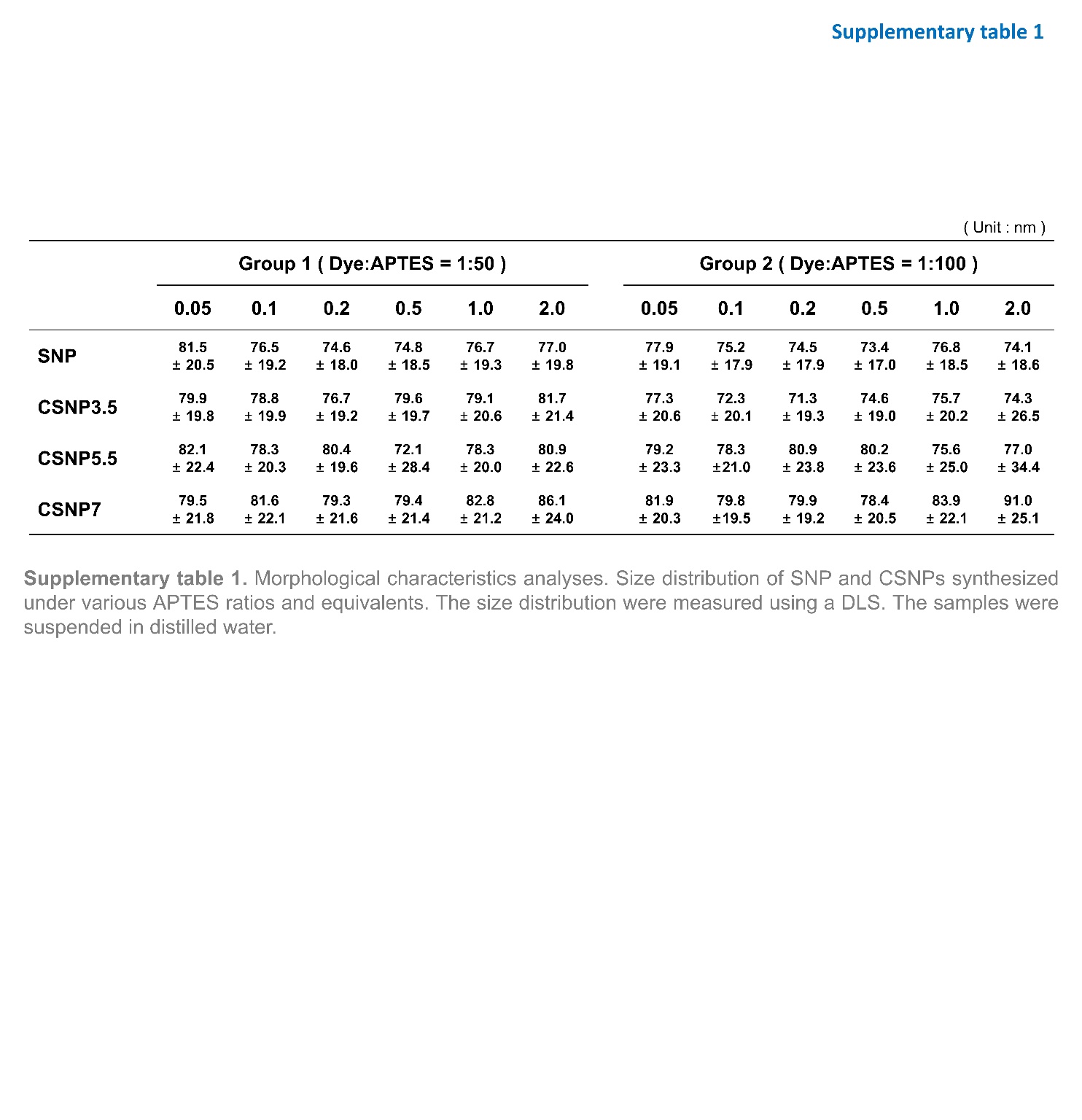


**Table S3.** Morphological characteristics analyses. Size distribution of SNP and CSNPs synthesized under various APTES ratios and equivalents. The size distribution were measured using a DLS. The samples were suspended in distilled water.


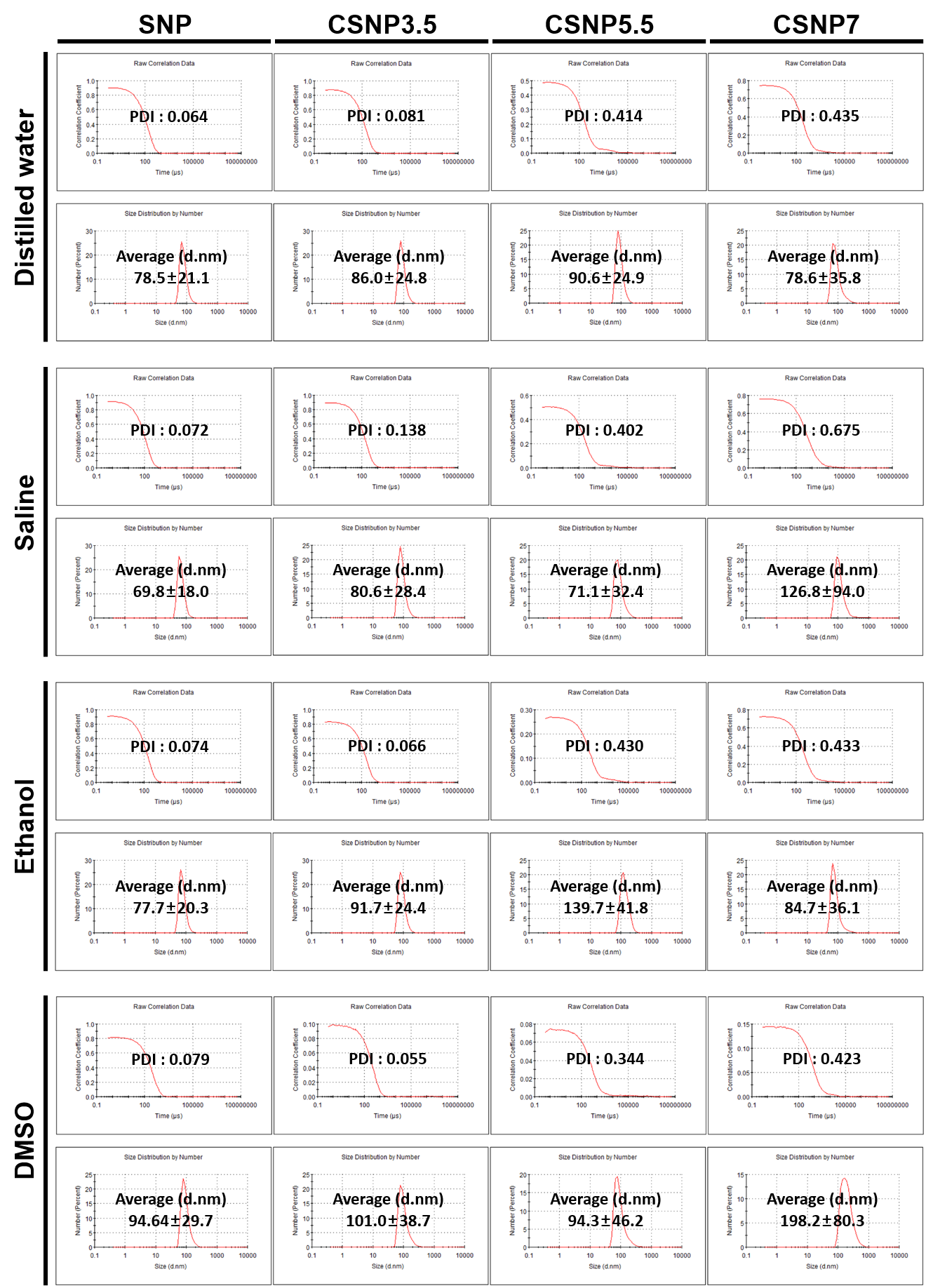


**Figure S4.** Confirmation of dispersibility of SNP and CSNPs in various solvents. The size distribution were measured by dynamic light scattering and quality reports were obtained from Zetasizer software. The samples were suspended in each solvent with 2 mg/ml concentration.


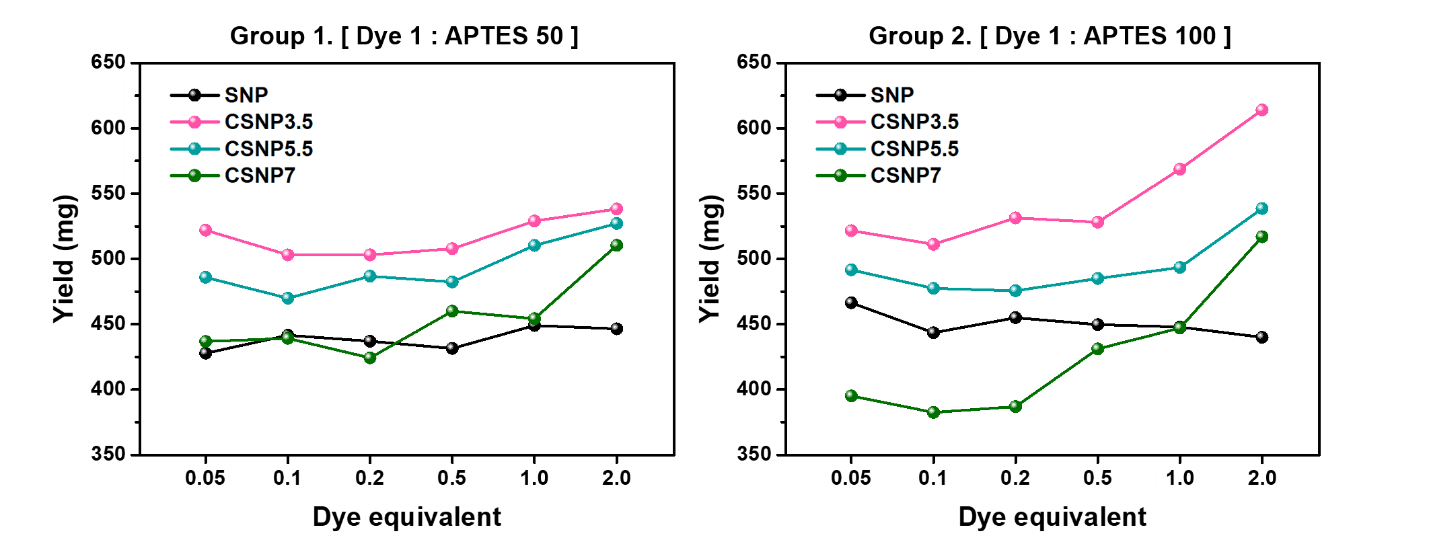


**Figure S5.** Yield of SNP and CSNPs synthesized under various APTES ratios and equivalents.


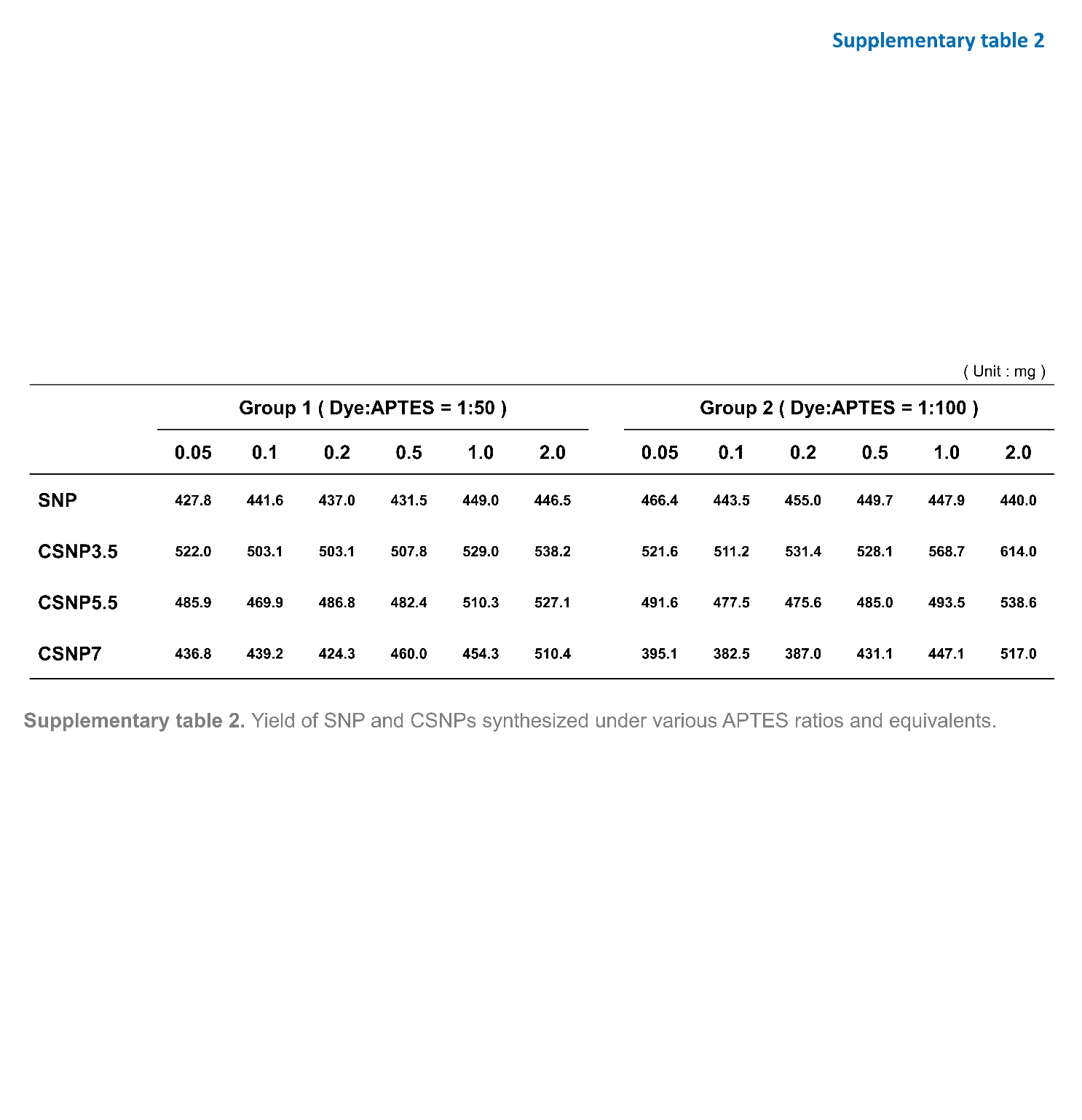


**Table S4.** Yield of SNP and CSNPs synthesized under various APTES ratios and equivalents.


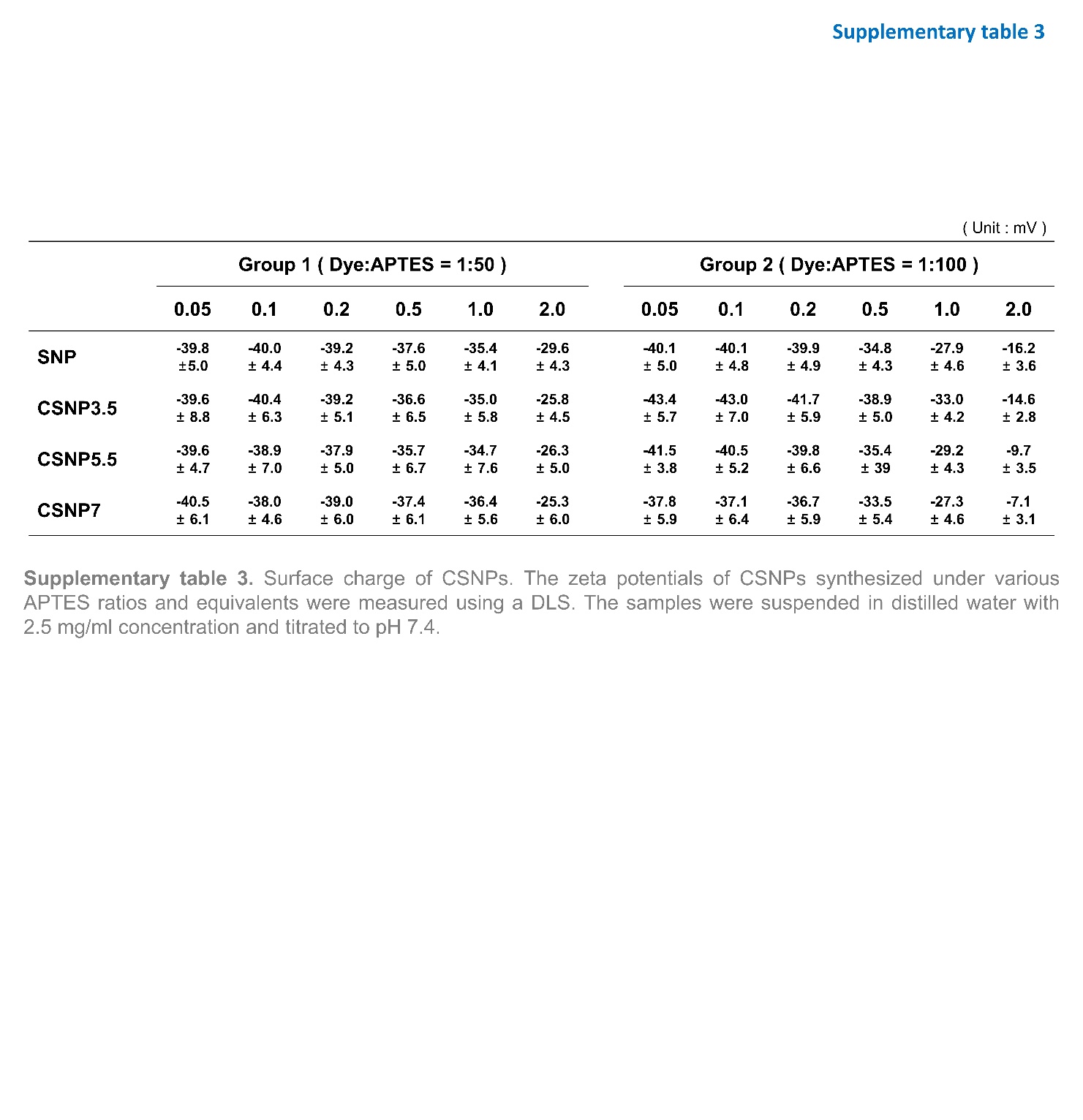


**Table S5.** Surface charge of CSNPs. The zeta potentials of CSNPs synthesized under various APTES ratios and dye equivalents were measured using a DLS. The samples were suspended in distilled water with 2.5 mg/ml concentration and titrated to pH 7.4.


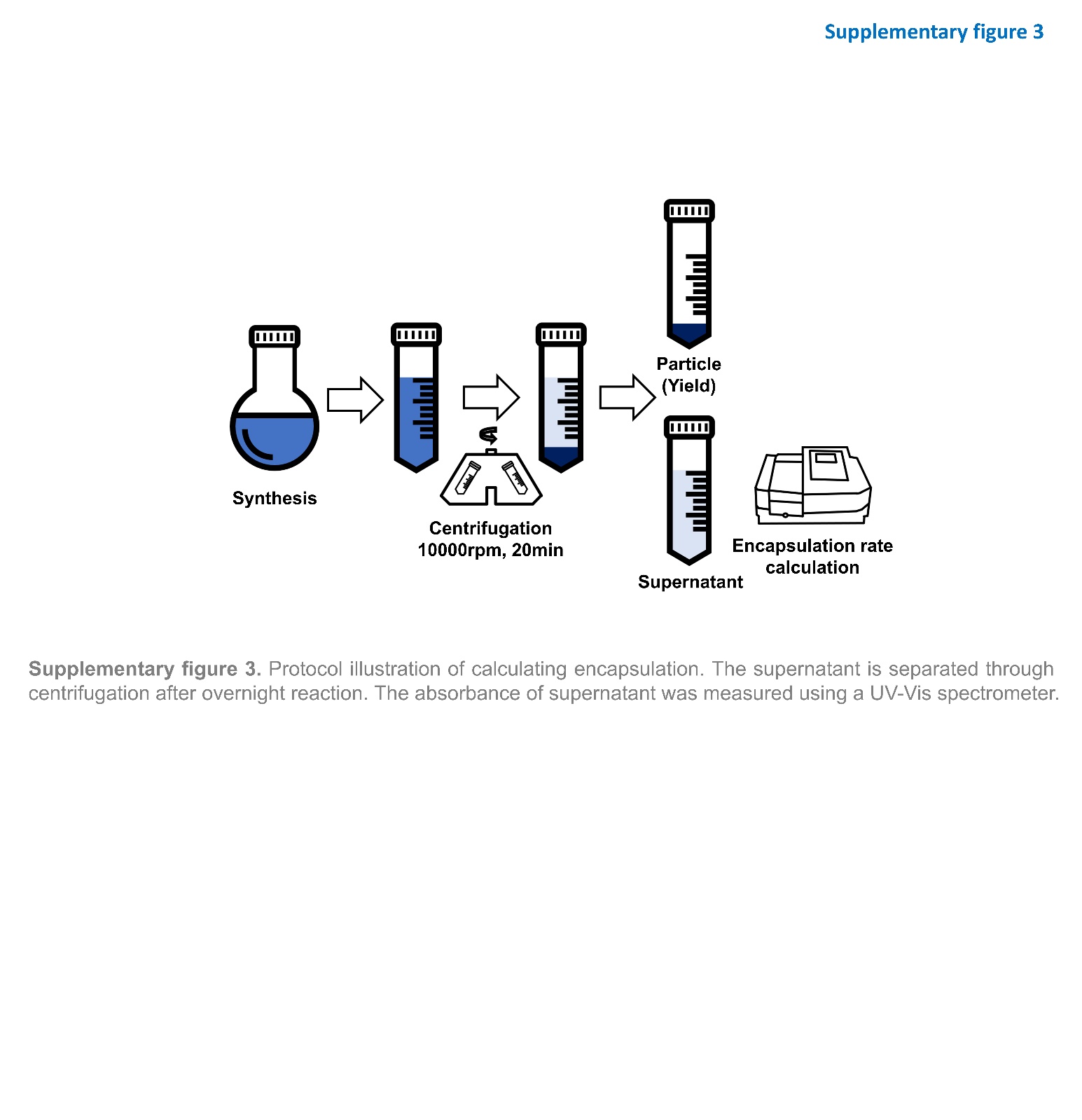


**Figure S6.** Protocol illustration of calculating encapsulation. The supernatant is separated through centrifugation after overnight reaction. The absorbance of supernatant was measured using a UV-Vis spectrometer.


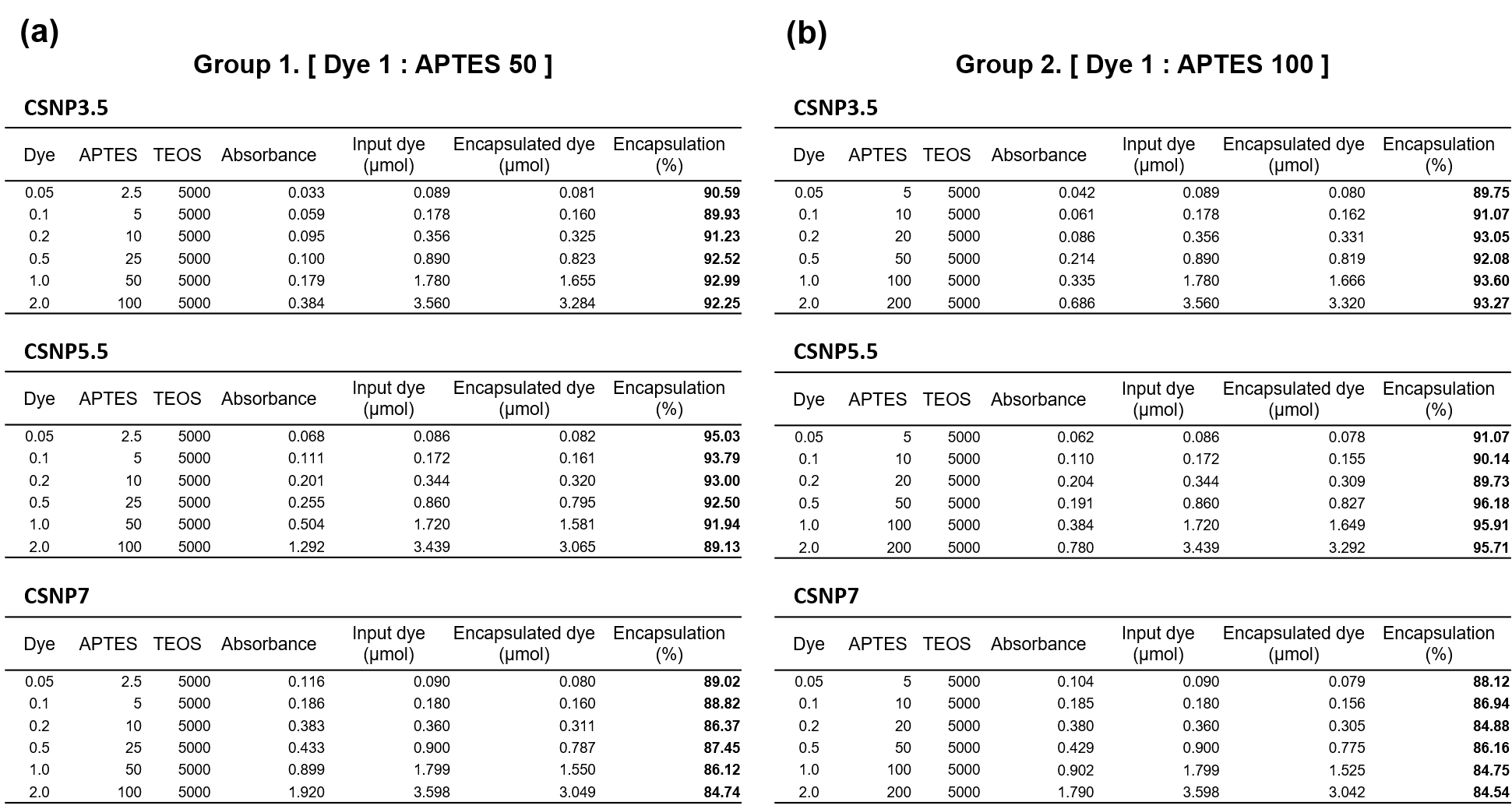


**Table S6.** Encapsulation of cyanine dyes in CSNP synthesized under various APTES ratios and equivalents. (a) Group synthesized with a 50-fold molar ratio excess of APTES to dye. (b) Group with a 100-fold. Initial addition to the reaction and encapsulated amount of dye for calculating encapsulation.


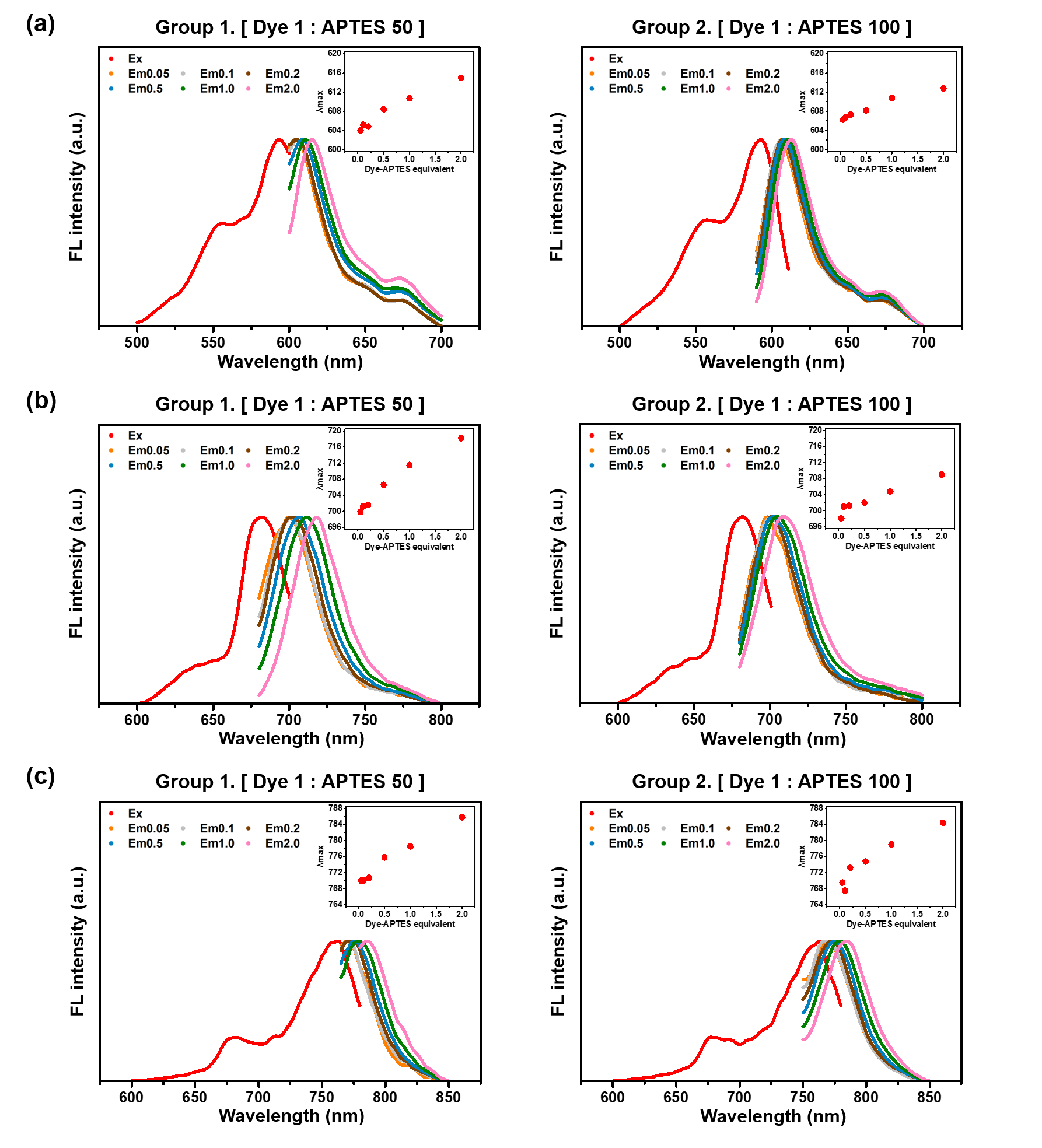


**Figure S7.** Emission spectra of CSNPs synthesized under various APTES ratios and equivalents were measured using a fluorescence spectrophotometer. (a) CSNP3.5, (b) CSNP5.5, (c) CSNP7. Group synthesized with a 50-fold molar ratio excess of APTES to dye (left) and with a 100-fold (right).


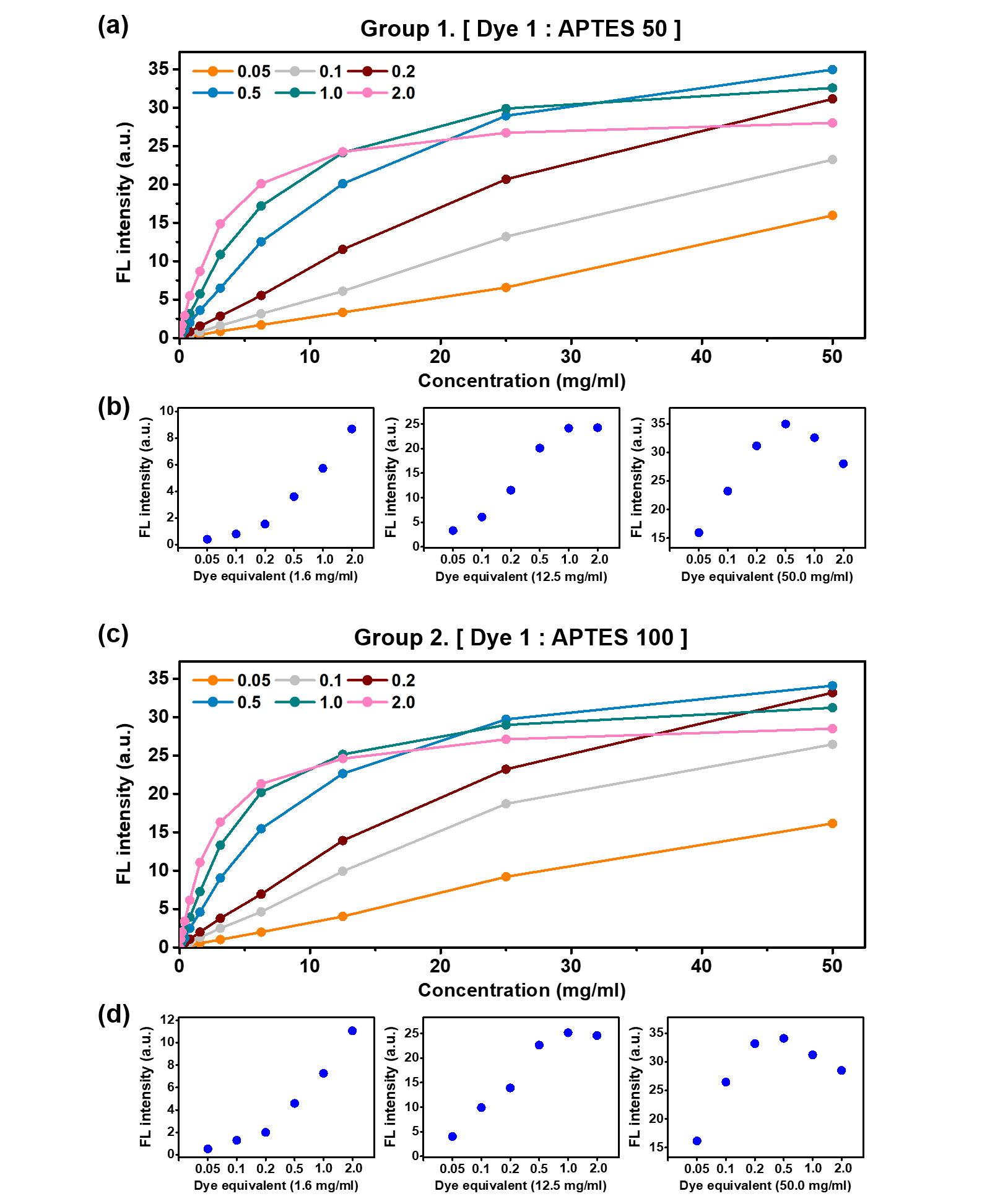


**Figure S8.** Comparison of fluorescent signal increase depending on the concentration of CSNP3.5 synthesized under various APTES ratios and equivalents. (a, b) Group synthesized with a 50-fold molar ratio excess of APTES to dye. (c, d) Group with a 100-fold. (a, c) Increase of fluorescence signal with concentrations ranging from 0 to 50 mg/ml. (b, d) Comparison of fluorescent signal efficiency at low, middle, and high concentrations. All signal intensities were obtained using a microplate reader.


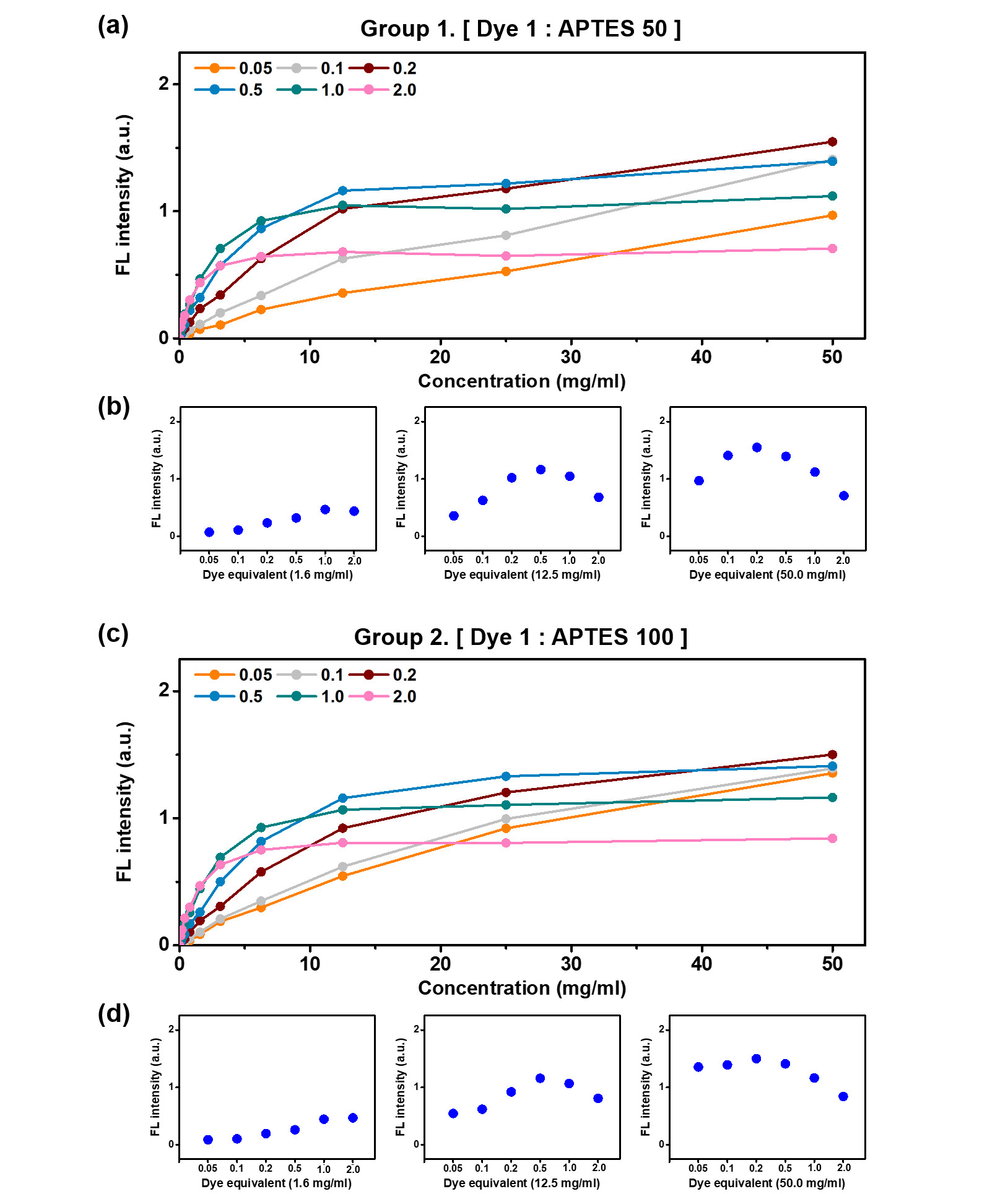


**Figure S9.** Comparison of fluorescent signal increase depending on the concentration of CSNP7 synthesized under various APTES ratios and equivalents. (a, b) Group synthesized with a 50-fold molar ratio excess of APTES to dye. (c, d) Group with a 100-fold. (a, c) Increase of fluorescence signal with concentrations ranging from 0 to 50 mg/ml. (b, d) Comparison of fluorescent signal efficiency at low, middle, and high concentrations. All signal intensities were obtained using a microplate reader.


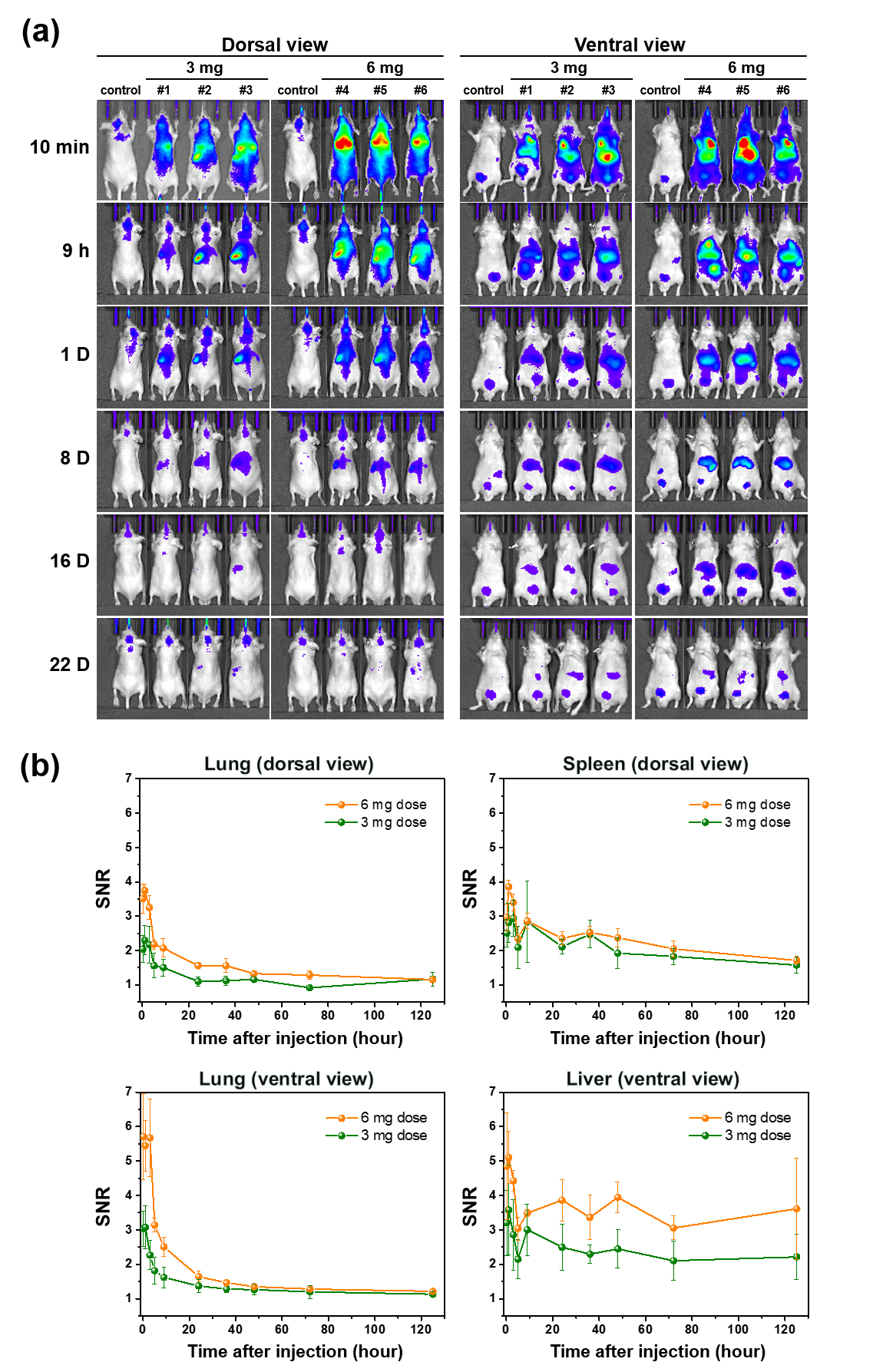


**Figure S10.** *In vivo* fluorescence imaging and biodistribution study of CSNP7. (a) Time-dependent *in vivo* fluorescence images of mice injected intravenously with CSNP7 at different doses (3 and 6 mg). (b) Signal-to-noise (SNR) obtained from the fluorescence intensities of major organs at different time points, shown from dorsal and ventral views.
